# A scoping review of the individual, socio-cultural, environmental and commercial determinants of gambling for older adults: implications for public health research and harm prevention

**DOI:** 10.1186/s12889-022-14930-y

**Published:** 2023-02-20

**Authors:** Rebecca H. Johnson, Hannah Pitt, Melanie Randle, Samantha L. Thomas

**Affiliations:** 1 Institute for Health Transformation, Faculty of Health, Deakin University, Locked Bag 20000, VIC 3220 Geelong, Australia; 2 School of Business, Faculty of Business and Law, University of Wollongong, Building 40, Northfields Ave, Wollongong, NSW 2500 Australia

**Keywords:** Gambling, Older adults, Scoping review, Determinants of health

## Abstract

Gambling is a global public health issue that can cause harm to individuals, families, and communities. Older adults are vulnerable to gambling harm due to life-stage experiences. This study aimed to examine current research relating to individual, socio-cultural, environmental, and commercial determinants of gambling among older adults. A scoping review was conducted (PubMed, PsycInfo, SocIndex, CINAHL Complete, Web of Science, Social Science and Sociology databases available in ProQuest, Google Scholar, citation searching), with peer reviewed studies included that were published between 1 December 1999 and 28 September 2022. Included studies were published in English in peer-reviewed journals that examined the determinants of gambling in adults aged 55 and over. Records were excluded if they were experimental studies, prevalence studies or had a population wider than the required age group. Methodological quality was assessed using JBI critical appraisal tools. Data was extracted using a determinants of health framework and common themes were identified. Forty-four were included. Most literature examined individual and socio-cultural determinants including reasons for gambling, risk management strategies, and social motivations for gambling. Few studies investigated environmental or commercial determinants, and those that did focused on accessibility of venues or promotions as pathways to gambling. Further research is needed to understand the impact of gambling environments and industry, and effective public health responses for older adults.

**Supplementary Information:**

The online version contains supplementary material available at 10.1186/s12889-022-14930-y.

## Introduction

Gambling is recognised as a neglected and understudied public health issue that has extensive consequences for the health and wellbeing of communities [1]. The rapid expansion of the industry, and the unknown adverse impacts of recent global events have left public health researchers recommending an urgent need to understand the range of complex factors that may lead to public health action to prevent gambling related health harms [1, 2]. Until recently, gambling has been viewed as an issue that is primarily related to individual behaviours and proclivities, and viewed from addictions-based psychiatric frameworks that have led to a predominant focus on gambling disorder [3]. However, public health perspectives highlight that the negative impacts of gambling go beyond the individual, and may be attributed to sociocultural factors, the environments in which gambling products are provided, and the commercial tactics to promote consumption [2, 4]. The wide-ranging harms caused by gambling impact gamblers at all risk levels, family members and communities [5]. Langham and colleagues ([6], p.4) define gambling related harm as:

“…any initial or exacerbated adverse consequence due to an engagement with gambling that leads to a decrement to the health or wellbeing of an individual, family unit, community or population”

Previous research has primarily focused on the individual determinants of gambling, including risk factors for problem gambling [7]. However, this individualised focus and ‘problem gambling’ rhetoric has led to a historically narrow scope for gambling research that lends itself to ‘victim blaming’ [8]. This has created a discourse whereby the burden of gambling harm is perceived to lie with individuals who are unable to control their gambling [9]. Additionally, focusing on the individual determinants of gambling leads to responses that involve relatively narrow strategies, such as changing or managing individual behaviour [3]. Until recently, the primary focus of gambling harm minimisation has involved responsible gambling approaches and tools. These strategies have been criticised for having modest effects because they address only individual factors and ignore broader structural issues associated with the addictive nature of gambling products, and their accessibility in community settings [3]. Existing evidence suggests that such approaches have been largely ineffective and that responsible gambling tools do not substantially lower the risk for problem gambling [10].

Hitherto, there has been little focus on the environmental and commercial determinants of gambling harm, and strategies that may address these. Such determinants include the nature of gambling products and promotions, and the characteristics of gambling environments that may appeal to different sub-populations. The Australian Productivity Commission [11] stated that understanding determinants of gambling beyond the individual are essential for developing public health responses to gambling. However, a lack of evidence has limited the design and implementation of comprehensive, population-based public health approaches to meet the needs of different population subgroups [2]. Adams and colleagues [12] propose that public health approaches to gambling must include interventions that shift the focus from individuals to the contexts and environments that drive behaviours. More recently, there have been additional concerns that the failure to expressly consider industry determinants in public health frameworks may deflect attention towards other social determinants:

“Whilst commercial causes of public health problems are ignored or obscured, thus omitting consideration of the need for closer regulation of harmful commodities and their producers” [[13], p.3].

There has been unprecedented growth in commercial gambling in recent decades and increased gambling related problems have emerged [14]. Calado and Griffiths [15] reported that national past year prevalence rates of problem gambling ranged from 0.1 to 6%. Gambling related harms are not isolated to those classified as ‘problem gamblers’ and are experienced across all levels of the risk continuum [16]. In fact, the majority of gambling related harm has been experienced by those other than ‘problem gamblers’ [17]. Gambling participation varies across the globe, 66.2% of Canadians reported engaging in some form of gambling in 2018 [18], around 35% of Australians aged 18 years and over spent money on gambling in a typical month (regular gamblers) in 2018 [19], and 44% of adults in the UK reported they were regular gamblers in the year to September 2022 [20]. Abbott [14] suggests that this continued growth of commercial gambling will expand gambling related harms into new population groups.

One population group identified as being particularly vulnerable to the environmental and commercial determinants of gambling harm are older adults (aged 55 years and above) [21]. Gambling participation rates among older adults vary across countries and range from 26.6 to 85.6% across national contexts [21]. Research also shows that in some countries, older adults have higher participation in gambling as compared to other population sub-groups. For example, in Australia, older adults are over-represented amongst regular gamblers with 30.4% of 50–64 year olds and 23.8% of adults aged 65 and over gambling at least monthly in 2015 [22]. The prevalence of problem gambling varies between countries, with estimates ranging from 0.01 to 10.6% in older adult populations [23].

A small number of prior reviews have sought to synthesise evidence related to older adults and gambling [13, 22–24]. Two of these reviews focused specifically on literature related to gambling disorders or problem gambling amongst older adults [23, 25]. Tse and colleagues [21] and Ariyabuddiphongs [24] had broader inclusion criteria and included studies about both problem gambling and recreational gambling. However, the findings from these reviews were also focused on disordered or problem gambling. Overall, such reviews have focused on the individual and socio-cultural determinants of problem gambling. They have suggested that factors such as loneliness, existing mental health issues, and a need for social connection may contribute to participation and vulnerability to harm. While this focus on individual and socio-cultural determinants is important, the reviews have not considered the broader range of environmental and commercial determinants that may influence older adults’ gambling attitudes and behaviours, the interplay across levels of determinants, and older adults’ conceptualisations of risk. Furthermore, an updated review would be important given indications that gambling has become increasingly normalised for older adults because of tactics of the gambling industry [21].

Older adults are an attractive demographic for the gambling industry due to their growing numbers and relatively large amounts of free time [21]. They may be attracted to gambling venues due to the free transport, cheap meals and social activity incentives, as well as perceptions that gambling venues are a ‘safe’ environment [26]. Some social agencies also facilitate and encourage gambling opportunities for older adults, with research indicating that gambling may be the single biggest social activity for retirement home residents [27]. Previous research has indicated that there is a lack of social measures and legislation to regulate the gambling industry using marketing strategies to target vulnerable people such as older adults [25].

The aim of this review was to provide an up-to-date map and framework for understanding the extent and limits of current research relating to the individual, socio-cultural, environmental, and commercial determinants of gambling in older adults. This paper extends prior reviews by examining available evidence regarding the determinants of gambling among older adults, while moving beyond studies which focus on ‘problematic’ gambling behaviour. The bulk of the extant review studies have focused on older adult problem gamblers. This is one of the few studies that examines older adults’ gambling more broadly, including those classified as non-problem gamblers. The analyses were guided by four main research questions:

1. How does the literature on older adults who gamble frame the individual, socio-cultural, environmental and commercial determinants of gambling?
2. What are the outcomes or recommendations from research conducted on older adults who gamble?
3. What are the gaps in knowledge regarding the determinants of gambling for older adults?
4. What is the methodological quality of the literature on older adults who gamble?

## Methods

The Preferred Reporting Items for Systematic Reviews and Meta-Analyses (PRISMA) was adhered to for the reporting of this review [28]. The protocol has not been registered for this review.

### Search strategy

A comprehensive search strategy was developed to identify peer reviewed studies relating to older adults and gambling that were published between 1 December 1999 and 28 September 2022. The search terms in Table 1 were used in EBSCOhost and ProQuest to search six online databases (CINAHL Complete, PubMed, PsycInfo, SocIndex and Web of Science in EBSCOHOST, and Social Science and Sociology databases in ProQuest), and identify potentially eligible records. These sources were last consulted on the 28 September 2022. The title and abstracts were screened by one author (RJ). The studies that were classified as included or unsure were then reviewed by all authors. The full-text articles of the included articles were then reviewed by one author (RJ), with studies classified as unsure subject to consensus from the remaining authors. Supplementary searches of the reference lists of included studies were also conducted, along with a search of Google Scholar using the terms ‘older adults and gambling’ and ‘elderly and gambling’ (the first five pages of ‘most relevant’ search hits were reviewed).

**Table 1:** Search terms

**Gambling Product Terms**	gambl* or lotter* or casino or “electronic gambling machine” or EGM or “sport* bet” or “sport* bets” or “sport* betting” or “horse* bet” or “horse* bets” or “horse* betting” or “online bet” or “online bets” or “online betting”
**Older Adult Terms**	“old* people*” or “old* adults*” or elder* or “late* life” or senior*

### Inclusion criteria and study selection

Studies were included if they used qualitative or quantitative methods and examined individual, socio-cultural, environmental or commercial determinants (definitions are provided at Table 2) of gambling in adults over 55 years of age. Articles were included if they were published in peer reviewed journals in the English language. The examination of abstracts, titles and full text articles led to the inclusion of 44 records. Experimental studies were excluded as they focused on clinical examinations of cognitive functioning of older adults which is outside the scope of this review about determinants. Studies involving samples of the general population were excluded as this study was interested in understanding the gambling of adults aged 55 years and over. Studies that only reported on the demographic characteristics of older adults who gamble were excluded from the review. Studies that reported on prevalence of gambling behaviour were excluded because these studies contained small cohorts of older adults and reported on prevalence data. Technical reports were not included in the review.

**Table 2:** Definitions of determinants of health used

**Individual determinants**: The individual determinants of health are comprised of an individual’s biological characteristics, their individual lifestyle behaviours and personal characteristics [29]. Note: This review will focus on the individual lifestyle behaviours of the individual determinants
**Socio-cultural determinants**: The socio-cultural determinants of health are how the context of one’s life can impact on their health. Socio-cultural factors include social networks, media, cultural traditions and customs that impact the health of individuals and communities [29]
**Environmental determinants**: The environmental determinants of health include factors within an individual’s environment that impact on their health, and in the context of gambling include the local gambling environment such as the availability and accessibility of venues [30, 31]
**Commercial determinants:** The commercial determinants of health are the market and non-market mechanisms utilised by the private sector that promote products and choices that adversely impact health such as marketing, lobbying, corporate social responsibility and extensive supply chains [13]

### Assessment of methodological quality

The critical appraisal of 44 included studies for methodological quality was subject to a single extraction by the primary author (RJ) using the JBI critical appraisal tools for qualitative [32] and cross-sectional designs [33]. Appropriate tools were used to assess the qualitative and quantitative sections of mixed methods studies. The critical appraisal was based on information in the published papers for 42 studies and the original methods papers were referred to for two studies including secondary data.

A grading system was used to rate the methodological quality of the papers. Studies were rated based on the following range of criteria met: low quality (0 to 33%); medium (34 to 66%); high quality (67% or more). Studies were not excluded based on their methodological quality. Supplementary table S1 shows the detailed results of the critical appraisal, with the results reported in narrative form.

### Data extraction and evidence synthesis

The data extraction was subject to single extraction by the primary author (RJ) with information extracted on the broad study characteristics including the country of origin and sample size; the study methodology including instruments or theoretical frameworks used; the relevant qualitative and/or quantitative data based on individual, socio-cultural, environmental or commercial determinants; the study recommendations; and the study funding sources. The data were compiled into a standardised data extraction template (see Supplementary table S2). The data were organised under the determinant categories: individual, socio-cultural, environmental and commercial. Common themes across the papers were identified, and the data synthesised into key themes. The key themes were reasons for gambling, gambling behaviour and expectations from gambling, perceptions of individual risk of gambling harm, strategies used to manage risks, social motivations, accessibility of gambling and gambling venues and promotions and incentives as pathways to venues. Finally, study recommendations were synthesised.

## Results

### Search results

**Fig. 1:**
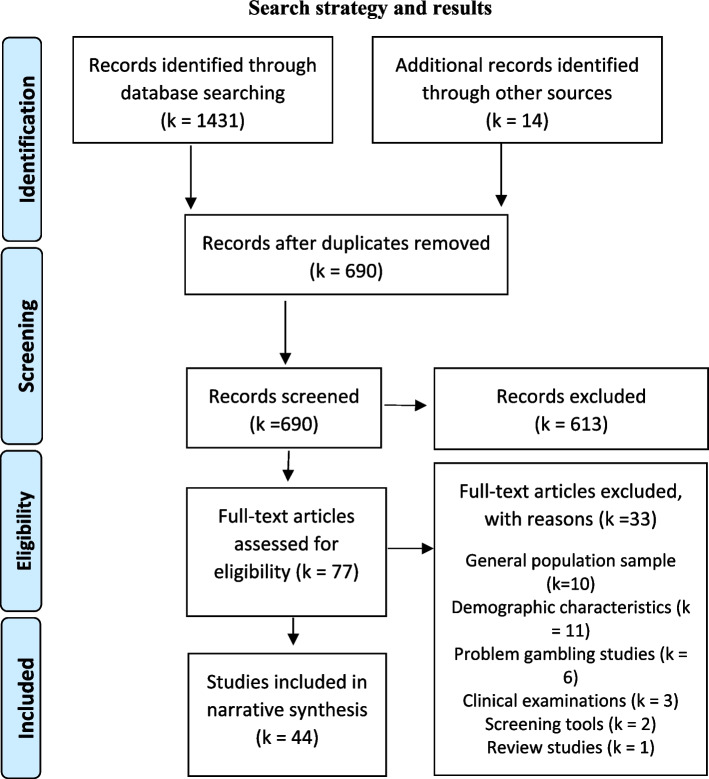
Search strategy and results

The initial keyword search yielded 1431 records. As shown in Fig. 1, 754 records were excluded in the first round because they were duplicates and 613 did not meet the inclusion criteria during abstract and title screening. Seventy-eight full text records were examined for eligibility and 34 were excluded because they reported only on the prevalence of problem gambling, profiled older adult gamblers, or included other population groups in the sample. A total of 44 records were included in the review (see Table 3 for an overview of characteristics of included studies; see Supplementary Table S2 for detailed results). The publication dates ranged from 2000 to 2022.

**Table 3:** Overview of the characteristics of each included study

Authors	Country	Sample size	Mean age (years)	Type of study	Determinants of gambling
Anderson, T.L, Rempusheski, V.F, & Leedy, K.N. (2018) [34]	United States	*n* = 34	m = 72	Qualitative	Individual Socio-cultural
Bazargan, M, Bazargan, S, & Akanda, M. (2001) [35]	United States	*n* = 80	m = 69	Quantitative	Individual
Bilt, J. V, Dodge, H. H, Pandav, R, Shaffer, H. J, & Ganguli, M. (2004) [36]	United States	*n* = 1016	m = 78.8, SD = 5.1	Quantitative	Individual Socio-cultural
Botterill, E, Gill, P. R, McLaren, S, & Gomez, R. (2016) [37]	Canada	*n* = 2103	m = 69.75, SD = 7.28	Quantitative	Individual Socio-cultural
Breen, H. (2009) [38]	Australia	*n* = 40	65 years and over	Qualitative	Individual Socio-cultural Environmental Commercial
Burge, A. N, Pietrzak, R. H, Molina, C. A, & Petry, N. M. (2004) [39]	United States	*n* = 52	m = 67, SD = 7	Quantitative	Individual Socio-cultural
Ciofi, J. (2019) [40]	United States	*n* = 13	Range: 65—92	Qualitative	Individual Socio-cultural Environmental Commercial
Clarke, D, & Clarkson, J. (2008) [41]	New Zealand	*n* = 104	m = 75.5, SD = 4.5	Quantitative	Individual Socio-cultural
Clarke, D, & Clarkson, J. (2009) [42]	New Zealand	*n* = 104	m = 74.5, SD = 4.5	Quantitative	Individual Socio-cultural
Elton-Marshall, T, Wiesingha, R, Sendzik, T, Mock, S. E, van der Maas, M, McCready, J, Mann, R.E, and Turner, N. E. (2018) [43]	Canada	*n* = 2103	Range: 55 – 75 +	Quantitative	Individual Socio-cultural
Hagen, B, Nixon, G & Solowoniuk, J. (2006) [44]	Canada	*n* = 12	m = 63.3	Qualitative	Individual Socio-cultural Environmental
Hillbrecht, M, & Mock, S. E. (2019) [45]	Canada	*n* = 3232	m = 63.7, SD = 5.34	Quantitative	Individual Socio-cultural
Hope, J, & Havir, L. (2002) [46]	United States	*n* = 168	Range: 60—85 +	Mixed methods	Individual Socio-cultural Environmental Commercial
Kim, W. (2020) [47]	United States	*n* = 14	Range: 65—89	Qualitative	Individual Socio-cultural Environmental
Kim, W, & Kim, S. (2020) [48]	United States	*n* = 20	m = 73, SD = 7.11	Qualitative	Individual Socio-cultural Environmental
Lelonek – Kuleta, B. (2021) [49]	Poland	*n* = 34	m = 65.3	Qualitative	Individual Socio-cultural
Lelonek – Kuleta, B. (2022) [50]	Poland	*n* = 34	m = 65.3	Qualitative	Individual Socio-cultural
Lelonek – Kuleta, B. (2022) [51]	Poland	*n* = 44	Range: 55—83	Qualitative	Individual Socio-cultural
Loroz, P.S. (2004) [52]	United States	*n* = 27	Range: 55—82	Qualitative	Individual Socio-cultural Environmental
Luo, H. (2021) [53]	Canada	*n* = 15	Range: 61—84	Qualitative	Individual Socio-cultural Environmental
McCarthy, S, Pitt, H, Bellringer, M.E. & Thomas, S.L. (2022) [54]	Australia	*n* = 20	m = 66, SD = 5.7	Qualitative	Individual Socio-cultural Environmental Commercial
Martin, F, Lichtenberg, P.A. & Templin, T.N. (2011) [55]	United States	*n* = 247	Range: 60 – 85 +	Quantitative	Individual Socio-cultural Environmental
McNeilly, D. P & Burke, W. J. (2000) [56]	United States	*n* = 315	m = 78.2, SD = 8.66	Quantitative	Individual Socio-cultural
Ng, V.C.K. (2011) [57]	Singapore	*n* = 74	m = 71.6, SD = 7.3	Quantitative	Individual Socio-cultural Environmental
O'Brien Cousins, S. O. B, & Witcher, C. (2004) [58]	Canada	*n = 17*	Range: 66—87	Qualitative	Individual Socio-cultural
O'Brien Cousins, S & Witcher, C. S. G. (2007) [59]	Canada	*n* = 444	m = 74.8	Quantitative	Individual Socio-cultural Commercial
Ohtsuka, K & Chan, C.C. (2014) [60]	Hong Kong	*n* = 18	Range: 55–85	Mixed methods	Individual Socio-cultural
Parekh, R & Morano, C. (2009) [61]	United States	*n* = 137	Range: 60 to 80	Mixed methods	Individual Sociocultural
Parke, A, Griffiths, M, Pattinson, J, & Keatley, D. (2018) [62]	United Kingdom	*n* = 595	m = 74.4	Quantitative	Individual Socio-cultural Environmental
Pattinson, J, & Parke, A. (2018) [63]	United Kingdom	*n* = 10	m = 70.4,	Qualitative	Individual Socio-cultural Environmental
Pattinson, J, & Parke, A. (2016) [64]	United Kingdom	*n* = 17	m = 76.82	Qualitative	Individual Socio-cultural Environmental
Penalba, E.H. (2020) [65]	Philippines	*n* = 2	Range: 80 +	Qualitative	Individual Socio-cultural Environmental
Phillips, W. J & Jang, S. (2012) [66]	United States	*n* = 681	Range: 65 +	Quantitative	Individual
Pitt, H, Thomas, S. L, Cowlishaw, S, Randle, M, & Balandin, S. (2022) [67]	Australia	*n* = 126	Range: 55 +	Qualitative	Individual Environmental
Southwell, J, Boreham, P, Laffan, W. (2008) [68]	Australia	*n* = 414	Range: 60 – 70 +	Mixed methods	Individual Socio-cultural Environmental
Subramaniam, M, Chong, S. A, Satghare, P., Browning, C. J, & Thomas, S (2017) [69]	Singapore	*n* = 25	m = 66.2 SD = 6.5	Qualitative	Individual Socio-cultural
Subramaniam, M, Satghare, P., Vaingankar, J. A., Picco, L, Browning, C. J, Chong, S. A, & Thomas, S (2017) [70]	Singapore	*n* = 25	m = 66.2 years SD = 6.5	Qualitative	Individual Socio-cultural
Thériault, É. R, Norris, J. E, & Tindale, J. A. (2018) [10]	Canada	*n* = 673	m = 68.78, SD = 7.6	Quantitative	Individual
Tira, C, & Jackson, A. C. (2015) [71]	Australia	*n* = 31	m = 67	Qualitative	Individual
Turner, N.E, van der Maas, M, McCready, J, Hamilton, H.A, Schrans, T, Ialomiteanu, A, Ferentzy, P, Elton-Marshall, T, Zaheer, S, & Mann, RE, (2018) [72]	Canada	*n* = 2103	Range: 55 to 75	Quantitative	Individual Environmenta Commercial
van der Maas, M, Hamilton, H.A, Matheson, F.I, Mann, R.E, Turner, N. E, and McCready, J. (2019) [73]	Canada	*n* = 2103	Range: 55 to 75 +	Quantitative	Individual
van der Maas, M, Mann, R. E, Matheson, F. I, Turner, N. E, Hamilton, H. A, & McCready, J. (2017) [74]	Canada	*n* = 2103	Range: 55 to 75 +	Quantitative	Individual Environmental
van der Maas, M, Mann, R. E, Turner, N. E, Matheson, F. I, Hamilton, H. A, & McCready, J. (2018) [75]	Canada	*n* = 2187	Range: 55 to 75 +	Quantitative	Individual Socio-cultural Commercial
Venuleo, C, Marinaci, T, & Mossi, P. (2021) [76]	Italy	*n* = 165	m = 66.9, SD = 5.7	Quantitative	Individual Socio-cultural

### Study characteristics

Most studies were conducted in the United States (k = 13), Canada (k = 12) and Australia (k = 5). Over half of studies (k = 21) were quantitative, with the majority (k = 16) of these being cross-sectional studies or secondary data analysis (k = 5) of cross-sectional studies. Three involved mixed methodologies, including quantitative surveys and semi-structured in-depth interviews. Twenty studies were qualitative. All studies focused on individual determinants (k = 44), and the majority also investigated the socio-cultural determinants (k = 35) of gambling behaviour. Sixteen studies examined an aspect of environmental determinants. Five quantitative studies and seven qualitative studies examined environmental determinants of gambling by investigating why older adults attend gambling venues. Seven qualitative studies also examined the environmental determinants of gambling by asking questions about how the gambling environment facilitated gambling. Seven qualitative studies and three quantitative studies investigated the commercial determinants of gambling by asking older adults about any promotions or bus tours offered by gambling venues.

Fifteen studies included a section that specifically focused on problematic or pathological patterns of gambling. Of these, four were secondary data analyses of quantitative data while nine were primary cross-sectional studies. The studies that included measures of problematic or pathological gambling examined concepts such as understanding older adults gambling behaviour, reasons for gambling, responsible gambling strategies used by older adults and the impact of psychosocial factors on problematic gambling. One qualitative study aimed to understand the motivations of women addicted to gambling and included a problem gambling screen. Another qualitative study involving older women who self-identified as being negatively impacted by gambling and examined how a range of factors impacted older women and their gambling. While a third qualitative study investigated whether males’ problem gambling severity varied with their motivation to gamble.

Twenty-four studies had no declared funding source, with the remainder funded by government, non-government, or charitable organisations (although it was unclear how many of these organisations were either directly or indirectly funded by money sourced from gambling taxation or revenue). One study included a declaration that an author had worked as a consultant for the gambling industry.

Fifteen qualitative studies in this review were rated as high quality and five were rated as medium quality (Supplementary Table S1). One of the mixed methods qualitative sections was rated as high quality while two were rated as medium quality. Lack of clarity for qualitative studies included not addressing the influence of the researcher on the research (Q7).

Fourteen of the 21 quantitative studies included in the review were rated as high quality and seven were rated as medium quality. Quantitative aspects of the mixed methods studies were rated as medium quality (k = 2) and low quality (k = 1). Aspects that were not addressed by all mixed methods studies included not measuring the independent variable in a valid or reliable way (Q3), not using a standard measurement of the condition (Q4) and not identifying (Q5) or dealing with confounding factors (Q6).

**Fig. 2:**
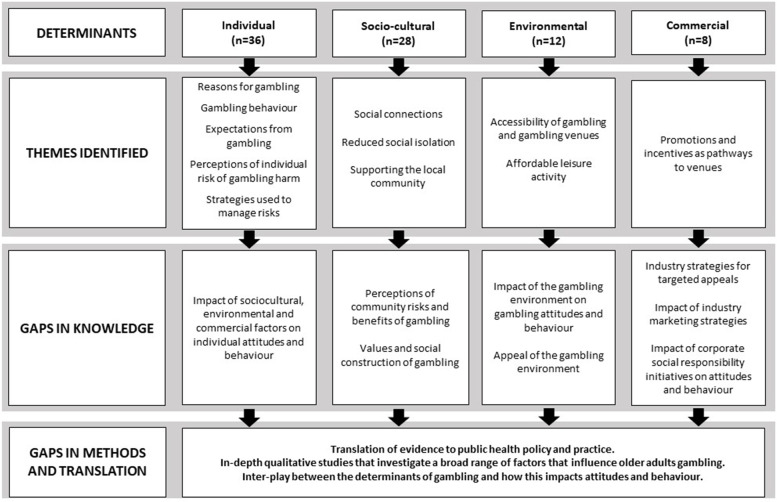
Findings of the review of literature on older adults gambling

The findings of the review are reported as the identified themes, under the categories of individual, socio-cultural, environmental, and commercial determinants. In addition, the gaps in knowledge, methods and translation as identified are reported. The broad findings of the review are graphically illustrated in Fig. 2.

### Individual determinants of gambling

#### Reasons for gambling

Extant studies indicated that older adults engaged in gambling for a variety of reasons. Three concepts emerged across studies: gambling as a form of entertainment, gambling to fulfil unmet psychological needs, and gambling to win money. Thirty papers described reasons why older adults engage in gambling. Results from several studies indicated that gambling was commonly regarded as fun and exciting. For example, in quantitative and qualitative studies from the United States, Canada and Poland, gambling was described as an exciting and fun form of entertainment [44, 51, 58 61]. Loroz [52] participants’ described gambling as a fun activity and were entertained by the fantasy of winning. Additionally, the participants in Penalba’s [65] study about gambling on cockfighting described gambling as a thrill-seeking pastime.

Multiple studies indicated that older adults engaged in gambling to fulfil unmet psychological needs. For example, a mixed methods Australian study found that one third (33%) reported gambling as a way of reducing depression or stress [30]. Quantitative studies and qualitative studies across a range of locations also found that gambling was used as an emotional escape for older adults [52–55, 63, 67, 69, 75]. Pattinson and Parke [64] developed this concept further with participants reporting they were using gambling as a temporary escape from bereavement, retirement, physical pain and physical deterioration. Building on this, Ciofi [40] reported that older adults used the casino as a site for maintaining feelings of self-sufficiency due to the casino being a site for active aging. A number of studies reported that older adults used gambling to meet the unmet psychological needs by enhancing their mood, increasing mental alertness or stimulation and using gambling to cope with difficult situations [38, 51, 52, 64]. Additionally older adults who were unpartnered and gambled to meet unmet psychological needs were more likely to experience problem gambling, particularly if they were divorced [43].

Some studies indicated that older adults perceived gambling to be a way of winning money [47, 52, 55, 65, 68]. For example, a study in the United States found that 63% went to casinos to win money, and 9% went to supplement their income [55]. Winning money was also given as a reason for gambling by 45% of participants in an Australian study with older adults who played Electronic Gambling Machines (EGMs) [68], also known as slots or poker machines. A study in the United States reported that older Chinese adults found gambling more enjoyable when they won money [47]. While a study on older males from Poland found that those experiencing problem gambling were more likely to gamble to win money [51].

#### Gambling behaviour and expectations from gambling

Some studies reported participants having expectations from their gambling, and that this impacted their behaviour. For example, some older adults gambled because they wanted to win money. A study in New Zealand found that the strongest reason for gambling was the financial rewards they received [41]. Other studies examined expectations of winning and gambling behaviours. For example, Phillips and Jang [66] conducted a survey-based quantitative study in the United States, and found that when older adults gambled to make money, they intended to gamble more than gamblers motivated by other reasons. However, in a study conducted in Canada, participants indicated that even though winning was not the main driver of playing they did expect that they would eventually win [58].

#### Perceptions of individual risk of gambling harm

Five studies explored how older adults conceptualise risk and harm. Studies from the United States, the United Kingdom and Australia found that very few older adults believed that they were at risk of gambling-related harm [46, 67, 63]. In their study of high frequency gambling with older females in the United Kingdom, Pattinson and Parke [63] found reduced perceptions of risks associated with gambling as compared to other addictive products. For example, participants indicated that there was no harm caused if an older person could afford to gamble and that gambling addiction impacts fewer people when compared to alcohol or drug addiction. Hagen and colleagues [44] conducted a study in Canada found that older adults considered gambling as a risky pastime, and many were able to recount stories of people they knew who had lost a lot of money gambling. However, Kim [47] reported that older Chinese adults in New York perceived they were not at risk due to betting small amounts and not having routines around gambling, while Pitt and colleagues [67] found that older adults felt they were not at risk due to employing responsible gambling strategies.

#### Strategies used to manage risks

Several studies examined how older adults managed the risks associated with gambling. A few studies [44, 52, 70] found that older adults described implementing a range of behavioural and cognitive strategies to manage and control their gambling behaviour. These included recognising that the odds were against them, restricting the amount of money available for gambling and delaying gratification which may have included not reinvesting their wins. Hagen and colleagues [44] reported that older adults managed their risks by playing games that cost less per game and gambling with friends. While Subramaniam and colleagues [70] reported older adults managed their risks of gambling harm by seeking help, maintaining balance in recreational activities and recognising disordered gambling in themselves and others.

Two other studies also reported that older adults used various strategies to limit the amount of money spent on gambling. For example, Hope and Havir [46] found, in the qualitative responses of their mixed methods study in the United States, that older adults used coupons and incentives to finance trips to casinos, and then limited the money they were allowed to spend on gambling. Pitt and colleagues [67] found that older Australian adults used time and monetary limits as risk management strategies. In a quantitative study in Canada, Theriault and colleagues [10] examined older adults’ use of responsible gambling strategies and the risk of problem gambling. They found no correlation between the number of responsible gambling strategies used and the scores on problem gambling measures.

### Socio-cultural determinants of gambling

#### Social connections

A range of studies examined the how older adults use gambling for social connection. Seven qualitative, one quantitative, and four mixed methods studies documented that many older adults engaged in gambling or attended gambling venues as a means of social connection [38, 40, 44, 46, 47, 51, 52, 60, 65, 68, 69, 73]. Five qualitative studies identified that older adults gambled due to the social interactions they are involved in while gambling [38, 40, 51, 52, 60] Similarly, in Singapore, Australia and the Philippines, researchers found that participants endorsed gambling as a social activity and indicated that gambling with family helped to foster familial connections [38, 65, 69]. Another Australian study revealed that participants used EGMs to ‘socialise and take others out’ or for a ‘social outing’ [68].

#### Reduced social isolation

Gambling was also used by older adults to reduce social isolation [38, 43, 53, 54, 58, 64, 68]. For example, McNeilly and Burke [27] and Pattinson and Parke [64], found that gambling was used by older adults to get out of the house and to decrease boredom. Similarly, in a study of 247 older adults, 38.2% of participants indicated they gambled to distract themselves from everyday problems such as loneliness and boredom, while 52.7% indicated that they gambled to be around other people [55]. Widowed participants in Elton Marshall and colleagues [43] study were significantly more likely to gamble due to loneliness. These results are further supported by an Australian study in which 34% of older adults gambled on EGMs to decrease isolation and 39% of adults gambled on EGMs to decrease boredom [68]. Additionally older adults used gambling to escape pressure or regular routines at home [38, 40, 52].

##### Supporting the local community

Two qualitative studies have documented that older adults engage in gambling as a way of supporting their local club and community [44, 58]. In a study in Canada, there were several participants that justified gambling due to the charitable activities that gambling companies engaged in [44]. The second study revealed that older adults perceived that Bingo helped the community. This was due to the belief that Bingo revenue is reinvested into community grants or activities for seniors [44, 58].

### Environmental determinants of gambling

#### Accessibility of gambling and gambling venues

Eleven studies identified that accessibility of gambling venues or activities was an enabling factor for older adults’ gambling. For example, studies conducted in the UK and the Philippines reported that older adults perceived that gambling was often available in convenient locations [62–65]. Additionally the older adults in these studies indicated that gambling was an accessible activity that older adults were able to engage in, when compared to other recreational activities [64], even if they were in poor health [62, 63]. Two Australian studies, and a study from the US reported that older adults attended gambling venues for a number of other reasons but gambled due to access to EGMs [67] or other gambling activities [38, 40]. A qualitative study in Canada, examined older women’s experiences of Bingo, and also found that Bingo was seen as physically and mentally accessible for women as they aged [58]. Numerous studies reported that older adults visited gambling venues because they were safe and inclusive spaces [46, 54, 62, 63].

#### Affordable leisure activity

Some studies have also reported that older adults may perceive gambling as an inexpensive and affordable leisure activity [38, 44, 59, 65]. For example a quantitative study in Singapore, indicated that lotteries were a popular, socially accepted form of gambling [57]. Playing the lottery was described as simple and accessible, with many locations that sold tickets, and inexpensive with tickets starting at $1 [57]. A study in the US reported that older adults placed a higher value on the money they used to gamble, due to the perceived high entertainment value of gambling [52]. Hagen and colleagues [44] study with non-problem gamblers, reported that older adults saw gambling as an activity that could facilitate an inexpensive holiday.

### Commercial determinants of gambling

#### Promotions and incentives as pathways to venues

Ten studies provided evidence to indicate that promotions and incentives for non-gambling products or activities impacted older adults’ gambling behaviour. For example, incentives such as affordable food, seniors’ meals, low-cost memberships, free transportation and other forms of non-gambling entertainment have been effective in creating pathways to gambling venues for older adults [38, 40, 44, 46, 53–55]. Southwell and colleagues [68] reported that older adults who participated in promotions offered by gambling venues spent more money gambling, while van der Maas and colleagues [74] identified increased rates of problem gambling among older adults who attend gambling venues for inexpensive food and beverages. A study in the US demonstrated that casinos offer promotions, gifts and gambling credits which encouraged older adults to attend the venue [40]. Two Australian studies reported that venues used strategies to encourage participation and to continue gambling on EGMs including free food and drinks, and the use of incentives [54, 67].

Some studies also examined how specific incentives provided by the gambling industry are linked to gambling behaviour and problems. For example, two quantitative studies in Canada examined combining gambling with tourism during casino bus tours. These studies revealed that older adults who engaged with these also tended to score more highly on problem gambling screens, when compared to those who do not engage with bus tours [72]. Additionally, bus tour participants were more likely to be problem gamblers with increased gambling behaviours and expenditure, particularly on EGMs [74].

### Study recommendations

The final phase of the analysis examined the recommendations proposed in the articles identified in the review. Thirty-four articles recommended that further research be conducted. The majority of the recommendations focused on the individual determinants, and suggested further research on the individual characteristics of older adult gamblers [34, 38, 51, 56, 63, 64, 70, 72] how gambling behaviours changed across lifetimes or age group cohorts [46, 55, 58, 61, 73] and that nationally representative quantitative studies were needed to understand gambling behaviours in older adults [35, 44].

Four articles included research recommendations related to socio-cultural determinants. This included measuring the costs of gambling behaviours to the community [42], focusing on the social nature of gambling activities for older adults [37, 45], and examining how a wider range of factors influence the psychosocial determinants of gambling [76]. Only two articles recommended investigating the role of commercial factors on older adults’ gambling. Southwell and colleagues [68] argued that research was needed to understand how the promotional tactics used by gambling venues influenced gambling behaviours in older adults while McCarthy and colleagues [54] recommended building upon their model for the commercial and political determinants of older women’s gambling. Two more studies mentioned that future research should seek to influence policy or regulation surrounding gambling [72, 74]. Suggestions included implementation of regulations requiring information about problem gambling provided to bus tour participants [72], and that policies could be implemented to regulate how gambling is marketed to older adults [75]. Pitt and colleagues [67] suggested that further research should explore older adults’ receptivity to information about gambling products and their associated harms.

Twenty-four studies made recommendations about future public health or health promotion action, with some papers making multiple recommendations. Many of these studies focused on developing individualised responsible gambling approaches to harm minimisation. The recommendations of studies focused on the individual older adult, by advocating for education initiatives that centred around appropriate messages for older adults, particularly about the impacts of problem gambling [35, 42, 44, 47, 48, 57, 61, 62, 70]. Another study suggested that it was important to understand which responsible gambling messages are appropriate for older adults [10]. Further recommendations focused on the individual harm minimisation with two studies suggesting increased training for primary care workers to identify gambling problems in older adults [42, 57]. Nine studies suggested the development of alternative recreational activities for older adults. For example, Botterill and colleagues [37] indicated that prevention initiatives should offer alternative social activities that aim to decrease loneliness and social isolation. Clarke and Clarkson [41] suggested older adults could engage in volunteer work to meet their social and entertainment needs. Other studies recommended that interventions or community organisations should focus on building older adults’ social networks, and cater for different groups of older adults (eg; women, different ethnicities) [38, 43, 45, 49, 50, 53, 54, 76].

Finally, four studies recommended addressing the commercial determinants of health as a public health response to gambling. Three studies recommended public education messaging about the harms of gambling products that are targeting towards older adults [50, 67] and older women [49]. Two Australian studies recommended reducing the availability and accessibility of EGMs in the community [67] and withdrawing the commercial factors that make gambling venues attractive for older women [54].

## Discussion

This review aimed to understand the available empirical literature regarding the individual, socio-cultural, environmental, and commercial determinants of gambling for older adults. This review identified 44 empirical studies that examined older adults gambling and found that older adults gambled for a variety of reasons, including to fulfil unmet psychological needs, to win money and for entertainment. They also gambled to support the community and decrease their isolation. Older adults were influenced to gamble by how accessible gambling was in their environment, while promotions and incentives offered by gambling venues are also pathways for them to gambling. Future research suggested by studies in this review focused on continuing to profile older adult gamblers and their characteristics. To reduce the chance of harm experienced by older adults from gambling, the studies in the review suggested education or harm messaging.

The review demonstrates that existing research on older adults has focused largely on examining the individual determinants of gambling. While the research identified in this review has examined individual and social reasons that older adults gamble (for example, to win money, socialise, and decrease isolation), the focus on individual determinants provides a narrow view of gambling for older adults. Gambling research has typically focused on the individual determinants of problem gambling [7], and this approach may reinforce the idea that the burden of gambling harm lies solely with those who are perceived to be unable to control their gambling [9]. Consequently, the strategies that are implemented to reduce harm are similarly narrow and tend to focus on managing individual behaviour [3]. These individual responsibility strategies have been criticised as being ineffective due to their focus on changing individual behaviour in the absence of consideration of wider mechanisms for reducing harm [3].

Previous research has indicated that environmental factors such as the accessibility and nature of gambling products needs to be considered to reduce gambling harm [3]. While the number of studies in this review that have examined the environmental and commercial determinants is limited, they provide evidence to suggest that the gambling environment and tactics of the gambling industry impact on older adults. Hellman and colleagues [77] argue that the industry effectively engages their clientele in different ways to gamble. For example, this review indicated that gambling venues are seen as accessible and safe places for older adults [54, 62, 63], and that promotions [55, 63] and incentives [67, 72] offered by the industry are appealing to older adults. No studies included in the review focused solely on the impact of environmental or commercial determinants of gambling on older adults. This is concerning given that international research has indicated that participation of older adults in gambling is increasing, and that this cohort is increasingly targeted with industry strategies [21].

The majority of studies included in this review recommended that further research should examine particular aspects of the individual determinants of gambling; for example individual characteristics of older adult gamblers, and individualised responses to harm minimisation. The continued referral of gambling research to investigate the individual determinants seems problematic as it does not support expansions of the evidence base to cover the broad range of factors that may influence older adults gambling. Gambling should be investigated from multiple viewpoints including the gambling environment and venue behaviour [78]. Without investigating the wide range of determinants of gambling for older adults, there will continue to be gaps in evidence and a lack of well-rounded responses to harm minimisation.

There are significant gaps in the research for all determinants of gambling for older adults, with the largest gaps in the environmental and commercial determinants (see Figure Two). The Australian Productivity Commission [79] previously indicated that understanding of how the range of determinants may shape gambling behaviour is integral to developing public health responses. However, limited research has investigated the environmental and commercial determinants of gambling for older adults. A recent study conducted by Thomas and colleagues [26] demonstrated that there are a range of environmental factors that draw older adults to gambling venues, including access to transport and parking, and that these venues are deemed safe. Even though the factors bringing individuals to the venue may not be associated with gambling, once in the venues people who attend regularly are more likely to participate in gambling activities [4, 54]. The inherent risks of gambling products at venues have been established, however little is understood about the impact of the gambling environment at these venues on older adults’ perceptions of risk and attitudes and behaviours. However, these drivers of gambling attitudes and behaviours have rarely been considered in research or policy responses. Researchers argue that public health responses to gambling need to focus on the context and environments that enable gambling behaviour [12, 78]. In fact, the omission of this broader range of determinants, including environmental and commercial determinants in public health approaches may inflate the importance of other determinants without considering the need for actions such as regulation of harmful products [13].

The present scoping review highlights that further research is required to understand the specific ways in which the gambling industry targets and appeal to older adults, and how this may encourage gambling to become a regular part of their lives. For example, research is required to examine how marketing strategies used by the industry impact the population of older adults. This will allow mapping and monitoring of how the gambling industry targets older adults and will enable appropriate public health measures and policy to be implemented.

The scoping review has a number of strengths due to the method followed. Firstly, the scoping review had transparent and rigorous methods. This included that the protocol was determined prior to conducting the review, the search strategy was systematic, and reproducible, and the data extraction and presentation occurred in a consistent manner [80]. This led to the production of a map of current knowledge, and the gaps in the research about older adults and their gambling. Secondly, this review incorporated a quality assessment of the included research. While this is not typical of a scoping review, it enabled the authors to report on the quality of, methods used, and the type of evidence that exists on older adults and gambling [80]. This allowed the identification of gaps in both the type and quality of the method of previous research conducted about older adults gambling.

## Limitations

There are several limitations that need to be considered. First, selection bias and cultural bias need to be considered as most studies that met the inclusion criteria were from North America. Investigating older adults’ gambling from different environmental and cultural perspectives will be important in understanding the wide variety of gambling experiences internationally. Second, studies may not have been included because they were not published in the English language, or because only six databases were searched. Third, only peer-reviewed studies were included in the review, and relevant information such as government reports and other grey literature were not included. An examination of grey literature including government reports could yield further information particularly about the various contexts that older adults gambling occurs in internationally. The review methodology did not exclude papers based on the rating of the methodological quality and data extraction and assessment of methodological quality was conducted by one author (RJ) which may lead to additional bias within the review. Additionally, the review protocol was not registered with a database prior to the review being conducted.

## Conclusions

This scoping review reveals that most research on older adults and gambling focuses on the individual determinants of gambling. This framing of older adults gambling around the individual determinants has led to a narrow understanding of the influences on older adults gambling behaviour and attitudes. It has also led to similarly narrow responses to minimise gambling harm. Additionally, the future research recommended by extant studies were focused on continuing to profile older adult gamblers by suggesting research focused on individual characteristics of older adults who gamble. Future health promotion or public health action recommended by included studies focused on further developing education strategies around gambling harm that are targeting older adults. This demonstrates that even with recommendations for future research, or public health action, there is still a focus on the individual determinants, without much consideration for the wider determinants of gambling among older adults. There is a need for further understanding of the impact of environmental and commercial determinants on older adults gambling. Future research needs to consider how gambling environments may influence older adults, how the industry may target older adult gamblers and appropriate public health measures that are likely to be motivating and effective for older adults.

## Supplementary Information

**Additional file 1:** **Supplementary Table S1.** Results of quality appraisal of methodological quality. 

**Additional file 2:** **Supplementary Table S2.** Summary of the included articles. 

**Additional file 3:** **Supplementary Table S3.** Table of papers excluded at full text, with reasons.

## Data Availability

All data generated of analysed during this study are included in this published article (and its supplementary information files).

## References

[1] The Lancet Public Health. Gambling: a neglected public health issue (Editorial). Lancet Public Health. 2021;6(1)e1. 10.1016/S2468-2667(20)30290-5. 10.1016/S2468-2667(20)30290-5 33417843

[2] Goyder E, Blank L, Baxter S, van Schalkwyk MCI (2019). Tackling gambling related harms as a public health issue. Lancet Public Health.

[3] Hancock L, Smith G (2017). Critiquing the Reno Model I-IV international influence on regulators and governments (2004–2015) - the distorted reality of “responsible gambling”. Int J Ment Health Addict.

[4] Bestman A, Thomas SL, Randle M, Pitt H, Daube M (2018). Attitudes towards gambling venues and support for regulatory reform: an online panel study of residents in New South Wales, Australia. Harm Reduct J.

[5] Abbott M, Romild U, Volberg R (2018). The prevalence, incidence, and gender and age-specific incidence of problem gambling: Results of the Swedish longitudinal gambling study (Swelogs). Addiction.

[6] Langham E, Thorne H, Browne M, Donaldson P, Rose J, Rockloff M (2016). Understanding gambling related harm: a proposed definition, conceptual framework, and taxonomy of harms. BMC Public Health.

[7] Young M (2012). Statistics, scapegoats and social control: a critique of pathological gambling prevalence research. Addict Res Theory.

[8] Van Schalkwyk M, Cassidy R, McKee M, Petticrew M (2019). Gambling control: in support of a public health response to gambling. Lancet.

[9] Miller HE, Thomas SL, Smith KM, Robinson P (2016). Surveillance, responsibility and control: an analysis of government and industry discourses about “problem” and “responsible” gambling. Addict Res Theory.

[10] Thériault ÉR, Norris JE, Tindale JA (2018). Responsible gambling strategies: Are they effective against problem gambling risk in older Ontarians?. J Gambl Issues.

[11] Australian Productivity Commission. Australia's Gambling Industries. Commonwealth of Australia. 1999. http://www.pc.gov.au/inquiries/completed/gambling/report/gambling1.pdf.

[12] Adams PJ, Raeburn J, De Silva K (2009). A question of balance: prioritizing public health responses to harm from gambling. Addiction.

[13] Maani N, Colin J, Friel S, Gilmore AB, McCambridge J, Robertson L, et al. Bringing the commercial determinants of health out of the shadows: a review of how the commercial determinants are represented in conceptual frameworks. Eur J Public Health. 2020;30(4):660–4. 10.1093/eurpub/ckz197 PMC7445044 31953933

[14] Abbott M. The epidemiology and impact of gambling disorder and other gambling-related harm. Discussion paper developed for the WHO forum on alcohol, drugs and addictive behaviours, 26–28 June 2017. https://www.who.int/docs/defaultsource/substance-use/the-epidemiology-and-impact-of-gambling-disorder-and-other-gambling-relate-harm.pdf?sfvrsn=5901c849_2.

[15] Calado F, Griffiths MD (2016). Problem gambling worldwide: an update and systematic review of empirical research (2000–2015). J Behav Addict.

[16] Miller H. Hidden harm: low-risk and moderate-risk gambling. Victorian Responsible Gambling Foundation; 2017. Available: https://responsiblegambling.vic.gov.au/documents/15/hidden-harm-low-and-moderate-risk-gambling.pdf.

[17] Browne M, Langham E, Rawat V, Greer N, Li E, Rose J, et al. Assessing gambling related harm in Victoria: a public health perspective. Melbourne: Victorian Responsible Gambling Foundation; 2016. https://responsiblegambling.vic.gov.au/resources/publications/assessing-gambling-related-harm-invictoria-a-public-health-perspective-69/.

[18] Williams RJ, Leonard CA, Belanger YD, El-Guebaly N, Hodgins DC, McGrath DS, Nicoll F, Stevens RMG (2021). Gambling and problem gambling in Canada in 2018: prevalence and changes since 2002. Can J Psychiatry.

[19] Australian Institute of Health and Welfare. Gambling in Australia 16 September 2021 Snapshot. Department of Health; 2021. Available: Gambling in Australia. https://www.aihw.gov.au/reports/australias-welfare/gambling.

[20] Gambling Commission. Findings from the quarterly telephone survey: Statistics on participation and problem gambling for the year to Sept 2022. UK Gambling Commission 2022. Available: Statistics on participation and problem gambling for the year to Sept 2022, October 25. https://www.gamblingcommission.gov.uk/statistics-and-research/publication/statistics-on-participationand-problem-gambling-for-the-year-to-sept-2022.

[21] Tse S, Hong SI, Wang CW, Cunningham-Williams RM (2012). Gambling behavior and problems among older adults: a systematic review of empirical studies. J Gerontol B Psychol Sci Soc Sci.

[22] Armstrong A, Carroll M. Gambling activity in Australia. Melbourne: Australian Gambling Research Centre. Australian Institute of Family Studies. 2017. https://aifs.gov.au/agrc/sites/default/files/publication-documents/rr-gambling_activity_in_australia_0.pdf.

[23] Subramaniam M, Wang P, Soh P, Vaingankar JA, Chong SA, Browning CJ, Thomas SA (2015). Prevalence and determinants of gambling disorder among older adults: a systematic review. Addict Behav.

[24] Ariyabuddhiphongs V (2012). Older adults and gambling: a review. Int J Ment Health.

[25] Guillou Landreat MG, Cholet J, Grail Bronnec M, Lalande S, Le Reste J (2019). Determinants of gambling disorders in elderly people – a systematic review. Front Psychiatry.

[26] Thomas S, McCarthy S, Pitt H, Daube M, Balandin S, Randle M, Cowlishaw S. Factors that shape the gambling attitudes and behaviours of older adults in Victoria, Victorian Responsible Gambling Foundation, Melbourne. 2020. https://responsiblegambling.vic.gov.au/resources/publications/factors-that-shape-the-gambling-attitudes-and-behaviours-of-older-adults-in-victoria-747/.

[27] McNeilly DP, Burke WJ (2001). Gambling as a social activity of older adults. Int J Aging Hum Dev.

[28] Page MJ, McKenzie JE, Bossuyt PM, Boutron I, Hoffmann TC, Mulrow CD (2021). The PRISMA 2020 statement: an updated guideline for reporting systematic reviews. BMJ.

[29] Australian Institute of Health and Welfare (AIHW). Australia’s health 2016. Australia’s health series no. 15. Cat. no. AUS 199. Canberra: AIHW. 2016. https://www.aihw.gov.au/reports/australias-health/australias-health-2016/contents/summary.

[30] Marshall D (2009). Gambling as a public health issue: the critical role of the local environment. J Gambl Issues.

[31] Thomas S, Pitt H, Bestman A, Randle M, McCarthy S, Daube M. The determinants of gambling normalisation: causes, consequences and public health responses, Victorian Responsible Gambling Foundation, Melbourne. 2018. https://responsiblegambling.vic.gov.au/documents/349/The_determinants_of_gambling_normalisation.pdf.

[32] Lockwood C, Munn Z, Porritt K (2015). Qualitative research synthesis: methodological guidance for systematic reviewers utilizing meta-aggregation. Int J Evid Based Healthc.

[33] Moola S, Munn Z, Tufanaru C, Aromataris E, Sears K, Sfetcu R, Currie M, Qureshi R, Mattis P, Lisy K, Mu P-F. Chapter 7: Systematic reviews of etiology and risk . In: Aromataris E, Munn Z (Editors). JBI Manual for Evidence Synthesis. JBI. 2020. Available from: https://synthesismanual.jbi.global.

[34] Anderson TL, Rempusheski VF, Leedy KN (2018). Casino gambling and the family: exploring the connections and identifying consequences. Deviant Behav.

[35] Bazargan M, Bazargan S, Akanda M (2001). Gambling habits among aged African Americans. Clin Gerontol.

[36] Bilt JV, Dodge HH, Pandav R, Shaffer HJ, Ganguli M (2004). Gambling participation and social support among older adults: a longitudinal community study. J Gambl Stud.

[37] Botterill E, Gill PR, McLaren S, Gomez R (2016). Marital status and problem gambling among Australian older adults: the mediating role of loneliness. J Gambl Stud.

[38] Breen H (2009). Senior citizen bingo players in australian registered and licensed clubs: a case study at tweed heads, New South Wales. J Travel Tour Mark.

[39] Burge AN, Pietrzak RH, Molina CA, Petry NM (2004). Age of gambling initiation and severity of gambling and health problems among older adult problem gamblers. Psychiatric Serv.

[40] Ciofi J (2019). Aging and personhood in the landscape of the mega-casino retirement at the tables. Anthropol Aging.

[41] Clarke D, Clarkson J (2008). Gambling behaviour and motivation in an urban sample of older adult gamblers. NZ J Psychol.

[42] Clarke D, Clarkson J (2009). A preliminary investigation into motivational factors associated with older adults’ problem gambling. Int J Ment Health Addict.

[43] Elton-Marshall T, Wijesingha R, Sendzik T, Mock SE, van der Maas M, McCready J, Mann RE, Turner NE (2018). Marital status and problem gambling among older adults: an examination of social context and social motivations. Can J Aging.

[44] Hagen B, Nixon G, Solowoniuk J (2006). Stacking the odds: a phenomenological study of non-problem gambling in later life. Can J Aging.

[45] Hillbrecht M, Mock SE (2019). Low-risk, moderate-risk, and recreational gambling among older adults: Self-complexity as a buffer for quality of life. App Res Qual Life.

[46] Hope J, Havir L (2002). You bet they're having fun!: older Americans and casino gambling. J Aging Stud.

[47] Kim W (2020). Healthy mahjong, little mahjong: social gambling among older Chinese immigrants in the U.S. Intl Gambl Stud.

[48] Kim W, Kim S (2020). “Gambling can’t be positive, can it?” Gambling beliefs and behaviors among older Korean immigrants. J Cross-Cultural Gerontol.

[49] Lelonek-Kuleta B (2021). Gambling and retirement: a qualitative study of non-problem gambling in the course of life of women. J Gambl Issues.

[50] Lelonek-Kuleta B (2022). Gambling motivation model for older women addicted and not addicted to gambling – a qualitative study. Aging Ment Health.

[51] Lelonek-Kuleta B (2022). Male gambling on retirement – qualitative analysis of problem and non-problem polish gamblers’ motivation to gamble. Int Gambl Stud.

[52] Loroz PS (2004). Golden-age gambling: Psychological benefits and self-concept dynamics in aging consumers’ consumption experiences. Psychol Mark.

[53] Luo H (2021). An exploratory study through a life course perspective: gambling among older Chinese people in a Canadian Context. Ageing Int.

[54] McCarthy S, Pitt H, Bellringer ME, Thomas SL. Electronic gambling machine harm in older women: a public health determinants perspective. Addict Res Theory. 2022. 10.1080/16066359.2021.1906864.

[55] Martin F, Lichtenberg PA, Templin TN (2011). A longitudinal study: casino gambling attitudes, motivations, and gambling patterns among urban elders. J Gambl Stud.

[56] McNeilly DP, Burke WJ (2000). Late life gambling: the attitudes and behaviors of older adults. J Gambl Stud.

[57] Ng VCK (2011). Gambling among older adults in Singapore some preliminary empirical findings. Asia Pac J Soc Work.

[58] O'Brien Cousins S, Witcher C. Older women living the bingo stereotype: “Well, so what? I play bingo I’m not out drinkin” I’m not out boozin’. Intl Gambl Stud. 2004;4(2):127–46.

[59] O’Brien Cousins S, Witcher CSG. Who plays bingo in later life? The sedentary lifestyles of “little old ladies.” J Gambl Stud. 2007;23(1):95–112. 10.1007/s10899-006-9030-8 17106654

[60] Ohtsuka K, Chan CC (2014). Senior gambling in Hong Kong: through the lenses of Chinese senior gamblers – an exploratory study. Asian J Gambl Issue Public Health.

[61] Parekh R, Morano C (2009). Senior gambling: Risk or reward?. J Gerontol Soc Work.

[62] Parke A, Griffiths M, Pattinson J, Keatley D (2018). Age-related physical and psychological vulnerability as pathways to problem gambling in older adults. J Behav Addict.

[63] Pattinson J, Parke A (2018). The experience of high-frequency gambling behavior of older adult females in the United Kingdom: an interpretative phenomenological analysis. J Women Aging.

[64] Pattinson J, Parke A (2016). Gambling behaviour and motivation in British older adult populations: a grounded theoretical framework. JGI.

[65] Penalba EH (2020). Cockfighting in later life: a qualitative inquiry into elderly Filipinos' gambling experiences. IRSSH.

[66] Phillips WJ, Jang S (2012). Exploring seniors’ casino gaming intention. J Hosp Tour Res.

[67] Pitt H, Thomas SL, Cowlishaw S, Randle M, Balandin S. “I always walked out with an empty purse”. Older adults’ engagement with electronic gambling machines in Victoria Australia. Health Promot J Austr. 2022;33:533. 10.1002/hpja.500. 10.1002/hpja.500 33982863

[68] Southwell J, Boreham P, Laffan W (2008). Problem gambling and the circumstances facing older people: a study of gaming machine players aged 60+ in licensed clubs. J Gambl Stud.

[69] Subramaniam M, Chong SA, Satghare P, Browning CJ, Thomas S (2017). Gambling and family: a two-way relationship. J Behav Addict.

[70] Subramaniam M, Satghare P, Vaingankar JA, Picco L, Browning CJ, Chong SA, Thomas SA (2017). Responsible gambling among older adults: a qualitative exploration. BMC Psychiatry.

[71] Tira C, Jackson AC (2015). Exploring the gray areas: senior gamblers’ perceptions of what is and what isn’t gambling. J Gambl Iss.

[72] Turner NE, van der Maas M, McCready J, Hamilton HA, Schrans T, Ialomiteanu, A, et al. Gambling behaviours and problem gambling among older adults who patronize ontario casinos or racinos. J Gambl Issues. 2018;(39 Special Issue):85–111. 10.4309/jgi.2018.39.4.

[73] van der Maas M, Hamilton HA, Matheson FI, Mann RE, Turner NE, McCready J (2019). Fun, money, and feeling down: Examining the associations between motivations and problem gambling among men and women in a sample of older adults. Int J Ment Health Addict.

[74] van der Maas M, Mann RE, Matheson FI, Turner NE, Hamilton HA, McCready J (2017). A free ride? An analysis of the association of casino bus tours and problem gambling among older adults. Addiction.

[75] van der Maas M, Mann RE, Turner NE, Matheson FI, Hamilton HA, McCready J (2018). The prevalence of problem gambling and gambling-related behaviours among older adults in Ontario. J Gambl Issues.

[76] Venuleo C, Tiziana M, Piergiorgio M (2021). Problem gambling among older people. An Italian study on habits, representations, levels off engagement and psychological determinants. J Gambl Issues.

[77] Hellman M, Cisneros Örnberg J, Livingstone C (2017). Gambling policy studies: a field that is growing in size and complexity. Addict Res Theory.

[78] Banks, G. Advancing the Reform Agenda: Selected Speeches. Productivity Commission. Canberra. https://www.pc.gov.au/news-media/speeches/reform-agenda.

[79] Australian Productivity Commission. (2010). Inquiry Report into Gambling. Commonwealth of Australia. 2012. Available: http://www.pc.gov.au/projects/inquiry/gambling-2009/report.

[80] Munn Z, Peters MDJ, Stern C, Trufanaru C, McArthur A, Aromataris E (2018). Systematic review or scoping review? Guidance for authors when choosing between a systematic or scoping review approach. BMC Med Res Methodol.

