# Supplementary material for: A scoping review of the individual, socio-cultural, environmental and commercial determinants of gambling for older adults: implications for public health research and harm prevention
Source: BMC Public Health. 2023 Feb 20;23:362. doi: 10.1186/s12889-022-14930-y (PMC9940406; doi:10.1186/s12889-022-14930-y)
Supplement: Supplementary file 1 — Additional file 1: Supplementary Table S1. Results of quality appraisal of methodological quality. [file 12889_2022_14930_MOESM1_ESM.pdf]

Supplementary Table S1: Results of quality appraisal of methodological quality

| Authors | Study Type | Raw Score on QA | Rating |
|---|---|---|---|
| Anderson, T.L, Rempusheski, V.F, and Leedy, K.N. (2017). | Qualitative | 9/10 | High |
| Breen, H. (2009). | | 5/10 | Medium |
| Ciofi, J. (2019). | | 9/10 | High |
| Cousins, S. O. B., & Witcher, C. (2004). | | 8/10 | High |
| Hagen, B, Nixon, G, & Solowoniuk, J. (2006). | | 7/10 | High |
| Kim, W. (2020). | | 8/10 | High |
| Kim, W, and Kim, S. (2020). | | 7/10 | High |
| Lelonek – Kuleta, B. (2021a). | | 5/10 | Medium |
| Lelonek – Kuleta, B. (2021b). | | 5/10 | Medium |
| Lelonek – Kuleta, B. (2022) | | 5/10 | Medium |
| Loroz. (2004). | | 8/10 | High |
| Luo, H. (2021). | | 7/10 | High |
| McCarthy, S, Pitt, H, Bellringer, M.E. & Thomas, S.L. (2021). | | 10/10 | High |
| Pattinson, J. and Parke, A. (2017). | | 10/10 | High |
| Pattinson, J. and Parke, A. (2017a). | | 9/10 | High |
| Penalba, E.H. (2020). | | 7/10 | High |
| Pitt, H, Thomas, S. L, Cowlishaw, S, Randle, M, & Balandin, S. (2021). | | 6/10 | Medium |
| Subramaniam, M, Chong, S. A, Satghare, P, Browning, C. J, & Thomas, S. (2017). | | 9/10 | High |
| Subramaniam, M, Satghare, P., Vaingankar, J. A., Picco, L, Browning, C. J, Chong, S. A, & Thomas, S (2017). | | 9/10 | High |
| Tira, C, & Jackson, A. C. (2015). | | 8/10 | High |
| | | | |
| Bazargan, M, Bazargan, S, & Akanda, M. (2001). | Cross sectional | 5/8 | Medium |
| Bilt, J. V, Dodge, H. H, Pandav, R, Shaffer, H. J, & Ganguli, M. (2004). | | 5/8 | Medium |
| Botterill, E, Gill, P. R, McLaren, S, & Gomez, R. (2016). | | 5/8 | Medium |
| Burge, A. N, Pietrzak, R. H, Molina, C. A, & Petry, N. M. (2004). | | 8/8 | High |
| Clarke, D, & Clarkson, J. (2008). | | 7/8 | High |
| Clarke, D, & Clarkson, J. (2009). | | 8/8 | High |
| Elton-Marshall, T, Wiesingha, R, Sendzik, T, Mock, S. E, van der Maas, M, McCready, J, Mann, R.E, and Turner, N. E. (2018). | | 6/8 | High |

| | | | |
|---|---|---|---|
| Hillbrecht, M, & Mock, S.E. (2019). | | 7/8 | High |
| Martin, F, Lichtenberg, P.A, & Templin, T.N. (2011). | | 6/8 | High |
| McNeilly, D. P, & Burke, W. J. (2000). | | 7/8 | High |
| Ng, V.C.K. (2011). | | 3/8 | Medium |
| O'Brien Cousins, S, & Witcher, C. S. G. (2007). | | 5/8 | Medium |
| Parke, A, Griffiths, M, Pattinson, J, & Keatley, D. (2018). | | 8/8 | High |
| Phillips, W. J, & Jang, S. (2012). | | 5/8 | Medium |
| Southwell, J, Boreham, P, & Laffan, W (2008). | | 7/8 | High |
| Thériault, É. R, Norris, J. E, & Tindale, J. A. (2018). | | 4/8 | Medium |
| Turner, N.E, van der Maas, M, McCready, J, Hamilton, H.A, Schrans, T, Ialomiteanu, A, Ferentzy, P, Elton-Marshall, T, Zaheer, S, & Mann, R.E, (2018). | | 6/8 | High |
| van der Maas, M, Mann, R. E, Matheson, F. I, Turner, N. E, Hamilton, H. A, & McCready, J. (2017). | | 6/8 | High |
| van der Maas, M, Mann, R. E, Turner, N. E, Matheson, F. I, Hamilton, H. A, & McCready, J. (2018). | | 7/8 | High |
| Van der Maas, M, Hamilton, H.A, Matheson, F.I, Mann, R.E, Turner, N. E, & McCready, J. (2019). | | 7/8 | High |
| Venuleo, C, Marinaci, T, & Mossi, P. (2021). | | 7/8 | High |
| | | | |
| Hope, J, & Havir, L. (2002). | Mixed methods | 4/10, 4/8 | Medium |
| Ohtsuka, K, & Chan, C.C. (2014). | | 8/10, 3/8 | High, medium |
| Parekh, R & Morano, C. (2009). | | 5/10, 2/8 | Medium, Low |

| Citation | Q1 | Q2 | Q3 | Q4 | Q5 | Q6 | Q7 | Q8 | Q9 | Q10 | Total | % |
|---|---|---|---|---|---|---|---|---|---|---|---|---|
| Anderson et al. 2017 | Y | Y | Y | Y | Y | Y | N | Y | Y | Y | 9/10 | 90[a] |
| Breen 2009 | N | Y | Y | Y | Y | N | N | U | U | Y | 5/10 | 50[b] |
| Ciofi 2019 | Y | Y | Y | Y | Y | Y | Y | Y | U | Y | 9/10 | 90[a] |
| Cousins & Witcher 2004 | Y | Y | Y | Y | Y | Y | N | Y | Y | U | 8/10 | 80[a] |
| Hagen et al. 2006 | Y | Y | Y | Y | Y | U | N | Y | U | Y | 7/10 | 70[a] |
| Hope & Havir 2002 | Y | Y | Y | U | Y | N | N | N | N | U | 4/10 | 40[b] |
| Kim 2020 | U | Y | Y | Y | Y | Y | N | Y | Y | Y | 8/10 | 80[a] |
| Ki & Kim2020 | U | Y | Y | Y | Y | N | N | Y | Y | Y | 7/10 | 70[a] |
| Lelonek-Kuleta 2021a | Y | Y | Y | U | U | N | N | N | Y | Y | 5/10 | 50[b] |
| Lelonek-Kuleta 2021b | Y | Y | Y | U | U | N | N | Y | N | Y | 5/10 | 50[b] |
| Lelonek-Kuleta 2022 | U | Y | Y | N | Y | N | N | U | Y | Y | 5/10 | 50[b] |
| Loroz 2004 | Y | Y | Y | Y | Y | N | Y | Y | U | Y | 8/10 | 80[a] |

| | | | | | | | | | | | |
|---|---|---|---|---|---|---|---|---|---|---|---|
| Luo 2020 | Y | Y | Y | Y | U | Y | N | N | Y | Y | 7/10 | 70[a] |
| McCarthy et al. 2021 | Y | Y | Y | Y | Y | Y | Y | Y | Y | Y | 10/10 | 100[a] |
| Ohtsuka & Chan 2014 | Y | Y | U | Y | Y | N | Y | Y | Y | Y | 8/10 | 80[a] |
| Parekh & Morano 2009 | U | Y | Y | U | Y | N | N | Y | U | Y | 5/10 | 50[b] |
| Pattinson & Parke 2017 | Y | Y | Y | Y | Y | Y | Y | Y | Y | Y | 10/10 | 100[a] |
| Pattinson & Parke 2017a | Y | Y | Y | Y | Y | Y | N | Y | Y | Y | 9/10 | 90[a] |
| Penalba 2020 | Y | Y | Y | Y | U | Y | N | Y | U | Y | 7/10 | 70[a] |
| Pitt et al. 2021 | U | U | Y | Y | Y | N | N | Y | Y | Y | 6/10 | 60[b] |
| Subramaniam et al. 2017 | Y | Y | Y | Y | Y | Y | N | Y | Y | Y | 9/10 | 90[a] |
| Subramaniam et al. 2017 | Y | Y | Y | Y | Y | U | N | Y | Y | Y | 8/10 | 80[a] |
| Tira & Jackson 2015 | Y | Y | Y | Y | Y | Y | N | N | Y | Y | 8/10 | 80[a] |

Y, yes; N, no; U, unclear
[a]High quality
[b]Medium quality
[c]Low quality
Q1 = Is there congruity between the stated philosophical perspective and the research methodology?
Q2 = Is there congruity between the research methodology and the research question or objectives?
Q3 = Is there congruity between the research methodology and the methods used to collect data?
Q4 = Is there congruity between the research methodology and the representation and analysis of data?
Q5 = Is there congruity between the research methodology and the interpretation of results?
Q6 = Is there a statement locating the researcher culturally or theoretically?
Q7 = Is the influence of the researcher on the research, and vice- versa, addressed?
Q8 = Are participants, and their voices, adequately represented?
Q9 = Is the research ethical according to current criteria or, for recent studies, and is there evidence of ethical approval by an appropriate body?
Q10 = Do the conclusions drawn in the research report flow from the analysis, or interpretation, of the data?

| **Citation** | **Q1** | **Q2** | **Q3** | **Q4** | **Q5** | **Q6** | **Q7** | **Q8** | **Total** | **%** |
|---|---|---|---|---|---|---|---|---|---|---|
| Bazargan et al. 2001 | Y | Y | Y | N | N | N | Y | Y | 5/8 | 63[b] |
| Bilt et al. 2004 | Y | Y | N | N | Y | Y | N | Y | 5/8 | 63[b] |
| Botterill et al. 2016 | Y | Y | N | Y | N | N | Y | Y | 5/8 | 63[b] |
| Burge et al. 2004 | Y | Y | Y | Y | Y | Y | Y | Y | 8/8 | 100[a] |
| Clarke & Clarkson 2008 | Y | Y | Y | Y | Y | Y | N | Y | 7/8 | 88[a] |

| | | | | | | | | | | |
|---|---|---|---|---|---|---|---|---|---|---|
| Clarke & Clarkson 2009 | Y | Y | Y | Y | Y | Y | Y | Y | 8/8 | 100[a] |
| Elton-Marshall et al. 2018 | Y | Y | N | N | Y | Y | Y | Y | 6/8 | 75[a] |
| Hillbrecht & Mock (2019) | Y | Y | N | Y | Y | Y | Y | Y | 7/8 | 88[a] |
| Hope & Havir 2002 | Y | Y | N | N | Y | N | N | Y | 4/8 | 50[b] |
| Martin et al. 2011 | Y | Y | N | Y | Y | Y | N | Y | 6/8 | 75[a] |
| McNeilly & Burke 2000 | Y | Y | Y | N | Y | Y | Y | Y | 7/8 | 88[a] |
| Ng 2011 | Y | Y | N | N | N | N | N | Y | 3/8 | 38[b] |
| O’Brien et al. 2017 | Y | Y | N | N | Y | Y | N | Y | 5/8 | 63 |
| Ohtsuka & Chan 2014 | Y | Y | N | N | N | N | Y | N | 3/8 | 38[b] |
| Parekh & Morano 2009 | Y | Y | N | N | N | N | N | N | 2/8 | 25[c] |
| Parke et al. 2018 | Y | Y | Y | Y | Y | Y | Y | Y | 8/8 | 100[a] |
| Phillips & Jang 2012 | Y | Y | N | N | Y | Y | N | Y | 5/8 | 63[b] |
| Southwell et al. 2008 | Y | Y | N | Y | Y | Y | Y | Y | 7/8 | 88[a] |
| Thériault et al. 2018 | Y | Y | N | N | N | N | Y | Y | 4/8 | 50[b] |
| Turner et al. 2018 | Y | Y | N | N | Y | Y | Y | Y | 6/8 | 75[a] |
| van der Maas et al. 2017 | Y | Y | N | N | Y | Y | Y | Y | 6/8 | 75[a] |
| van der Maas et al. 2018 | Y | Y | N | Y | Y | Y | Y | Y | 7/8 | 88[a] |
| van der Maas et al. 2019 | Y | Y | N | Y | Y | Y | Y | Y | 7/8 | 88[a] |
| Venuleo et al. 2021 | Y | Y | Y | N | Y | Y | Y | Y | 7/8 | 88[a] |

Y, yes; N, no; U, unclear
[a]High quality
[b]Medium quality
[c]Low quality
JBI critical appraisal checklist for analytical cross sectional studies
Q1 = Were the criteria for inclusion in the sample clearly defined?
Q2 = Were the study subjects and the setting described in detail?
Q3 = Was the exposure measured in a valid and reliable way?
Q4 = Were objective, standard criteria used for measurement of the condition?
Q5 = Were confounding factors identified?
Q6 = Were strategies to deal with confounding factors stated?
Q7 = Were the outcomes measured in a valid and reliable way?
Q8 = Was appropriate statistical analysis used?
