# Supplementary material for: A scoping review of the individual, socio-cultural, environmental and commercial determinants of gambling for older adults: implications for public health research and harm prevention
Source: BMC Public Health. 2023 Feb 20;23:362. doi: 10.1186/s12889-022-14930-y (PMC9940406; doi:10.1186/s12889-022-14930-y)
Supplement: Supplementary file 2 — Additional file 2: Supplementary Table S2. Summary of the included articles. [file 12889_2022_14930_MOESM2_ESM.pdf]

Supplementary Table S2: Summary of the included articles.

| **Authors** | **Aims and Research Questions** | **Determinants of gambling investigated** | **Country** | **Study Methodology** | **Sample** | **Key findings** | **Recommendations** | **Funding Sources** |
|---|---|---|---|---|---|---|---|---|
| Anderson, T.L, Rempusheski, V.F, & Leedy, K.N. (2018). | *Aim*: To investigate the relationship between gambling and family consequences.<br><br>*RQs*: No research questions. | Individual Socio-cultural | United States | In-depth qualitative interviews using Grounded Theory. Utilised maximum variation sampling and recruited from senior centres. | 62-88-year olds (n=34). | Gambling was influenced by family member participation in gambling activities. False expectations of the outcomes of gambling led to stress, tension and relationship issues. Participants indicated that their gambling had led to family discord. There was evidence that gambling had led to financial hardship. | *Future research*: Investigate how the interaction of innate personal characteristics and socio-cultural influences lead to behaviours. | None declared. |
| Bazargan, M, Bazargan, S, & Akanda, M. (2001). | *Aim*: To expand upon the current knowledge of elderly gambling behaviours, correlates and risk factors.<br><br>*RQs*: No research questions. | Individual | United States | A cross sectional study collected quantitative data through face to face interviews. | 60 - 91 year old African Americans (n=80). | Proportion of sample identified as non- or occasional gamblers (64%); light to moderate gamblers (19%); heavy to pathological gamblers (17%).<br>Analysis of data found a statistically significant relationship between stressful life events and gambling behaviour. Lower levels of well-being, higher anxiety and higher levels of OCD were found in heavy to pathological gamblers. | *Future research*: Conduct nationally representative studies on gambling.<br>*Future health promotion activities:* educate older people about potential impacts of PG.<br>*Future public health:* train primary care staff to be able to identify issues related to PG. | None declared. |

| Authors | Aims and Research Questions | Determinants of gambling investigated | Country | Study Methodology | Sample | Key findings | Recommendations | Funding Sources |
|---|---|---|---|---|---|---|---|---|
| Bilt, J. V, Dodge, H. H, Pandav, R, Shaffer, H. J, & Ganguli, M. (2004). | *Aim:* To examine the relationships between leaving the house to engage in gambling, and physical, mental health and social support among the elderly.<br><br>*RQs:* No research questions. | Individual<br>Socio-cultural | United States | Quantitative data was collected during a prospective epidemiological study. | 71 - 97-year olds from a low SES area (n=1016). | Younger age, greater social support, and alcohol use in the past year were independently associated with gambling activity. Longitudinally, age, sex, social support, alcohol use, and gambling are predictive of future gambling activity. | None. | National Institute of Aging US Department of Health and Human Services. |
| Botterill, E, Gill, P. R, McLaren, S, & Gomez, R. (2016). | *Aim*: To determine if motivations for gambling and going to the casino with others mediates the relationship between marital status and PG.<br><br>*RQs:* No research questions. | Individual<br>Socio-cultural | Canada | Secondary data analysis of quantitative data. | 55 years and over who attended casinos/ racinos (n=2103). | Single older adults were more likely to have higher levels of problem gambling, gamble alone, and gamble due to loneliness. The relationship between marital status and gambling was mediated by motivation for gambling when the motivation for gambling with others was to decrease loneliness. | *Future research:* Determine the role of family and friends on older adults gambling behaviour.<br>*Future health promotion/public health action:* Problem gambling may be prevented by addressing causes for gambling such as decreasing loneliness and social isolation among older adults. | None declared. |

| Authors | Aims and Research Questions | Determinants of gambling investigated | Country | Study Methodology | Sample | Key findings | Recommendations | Funding Sources |
|---|---|---|---|---|---|---|---|---|
| Breen, H. (2009). | *Aim:* To investigate the motivations and consequences of playing bingo by senior citizens in Australian Clubs.<br>*RQs:* No research questions. | Individual<br>Socio-cultural<br>Environmental<br>Commercial | Australia | Qualitative study using focus groups with older adults. | 65 years and over who attended Australian clubs (n=40). | Main themes surrounding motivation to gamble were socialisation, to decrease isolation and loneliness, to improve mood, to escape pressure at home, improve mental alertness and health, and due to it being a value for money activity. The participants attended the club for a number of reasons, including both gambling and non-gambling activities, multiple times per week. Some attended with family members. | *Future research:* A need for increased awareness of the gambling activities of older adults given increasing numbers of older adults retiring and the current increased availability and accessibility of gambling.<br>*Future health promotion/public health action:* Clubs and their buses could be a site for health and welfare agencies to make contact with older adults. Clubs could change their operations to meet older adults need for a sense of belonging. | None declared. |

| Authors | Aims and Research Questions | Determinants of gambling investigated | Country | Study Methodology | Sample | Key findings | Recommendations | Funding Sources |
|---|---|---|---|---|---|---|---|---|
| Burge, A. N, Pietrzak, R. H, Molina, C. A, & Petry, N. M. (2004). | *Aim:* To examine the relationship between age at first gambling experience and severity of gambling and related problems among older adult problem gamblers.<br><br>*RQs:* No research questions. | Individual<br>Socio-cultural | United States | Quantitative analysis of a self-reported problem gambling, addiction and health survey. | 60 - 74-year-old problem gamblers who had gambled at least 3 times per month (n= 52). Excluded if had major psychiatric disorder. | The median age of first gambling was 21 years. Those with an early onset gambled more frequently and had more severe medical and psychiatric problems.<br>In the multivariate analysis, age at gambling initiation was significantly associated with Addiction Severity Index score. | None. | National Institutes of Health and Patrick and Catherine Weldon Donaghue Medical Research Foundation Investigator Program. |
| Ciofi, J. (2019). | *Aim:* Focus on how mega casinos facilitate a variety for seniors including gambling.<br>*RQs:* No research questions. | Individual<br>Socio-cultural<br>Environmental<br>Commercial | United States | Ethnographic study of older adults who frequent mega casinos. | 65 – 92 year olds (n=13) who attend the casino at least weekly. | Casinos were seen as a site for active aging for older adults particularly once they retire from work. Casinos were able to provide a place for social interaction, mental stimulation and a sense of value for older adults. Additionally casinos provided activities adjacent to promotions, gifts or gambling credits which gave older adults reason to go out of the house for recreational activities. The casinos in this study also became a site for older adults to maintain their self sufficiency and manage their own schedules where they could attend to errands, have social interactions and participate in recreational activities. | None. | None declared. |

| **Authors** | **Aims and Research Questions** | **Determinants of gambling investigated** | **Country** | **Study Methodology** | **Sample** | **Key findings** | **Recommendations** | **Funding Sources** |
|---|---|---|---|---|---|---|---|---|
| Clarke, D, & Clarkson, J. (2008). | *Aim*: To compare the behaviour and motivation of 41 male and 63 female gamblers in Hamilton, New Zealand where a casino was recently opened.<br><br>*RQs*: No research questions.<br><br>*Hypotheses:* Five hypotheses focused on predictions of preferred gambling activity and motivations based on gender, that outcomes such as external rewards will be stronger motivators and regular gamblers will have higher expenditure and gamble to be stimulated. | Individual Socio-cultural | New Zealand | Cross sectional self-report survey and quantitative analysis. | 66 to 87 years of age who gambled at least once in past 12 months (n=104). | The motivation for gambling most frequently reported were rewards and boredom. Regular continuous gamblers had significantly higher scores on curiosity, stimulation, escape and apathy as motivation for gambling. | *Future research*: Longitudinal and observational studies should occur in locations with new casinos and large populations of older adults.<br><br>*Future public health action:* implement problem gambling screens with health professionals.<br>*Future health promotion action:* educate older adults about risks of PG and encourage alternative activities. | None declared. |

| **Authors** | **Aims and Research Questions** | **Determinants of gambling investigated** | **Country** | **Study Methodology** | **Sample** | **Key findings** | **Recommendations** | **Funding Sources** |
|---|---|---|---|---|---|---|---|---|
| Clarke, D, &Clarkson, J. (2009). | *Aim:* No aim.<br><br>*RQs:* No research questions.<br><br>*Hypotheses:* Four hypotheses focused on motivation differences between men and women, that particular internal and external motivations would be associated with problem gambling, that characteristics of an individual would impact on problem gambling outside of situational factors and that EGM and bingo players have higher problem gambling scores. | Individual<br>Socio-cultural | New Zealand | Cross sectional self-report survey and quantitative analysis. | 65 - 85+ years olds, who gambled for money (n=104). | Frequency of gambling, number of activities, largest amount spent per session and parents' gambling were significantly associated with gambling.<br>Stimulation and amotivation were motivational predictors of problem gambling.<br>Gambling for rewards and social recognition were not significantly associated with PG. | *Future research:* To examine the costs and impacts of older adults gambling in communities. | None declared. |

| Authors | Aims and Research Questions | Determinants of gambling investigated | Country | Study Methodology | Sample | Key findings | Recommendations | Funding Sources |
|---|---|---|---|---|---|---|---|---|
| Elton-Marshall, T, Wiesingha, R, Sendzik, T, Mock, S. E, van der Maas, M, McCready, J, Mann, R.E, and Turner, N. E. (2018). | *Aim:* Examine how gambling motivation and the social context can mediate the relationship between marital status and problem gambling status. *RQs:* no research question. | Individual Socio-cultural | Canada | Secondary analysis of quantitative date | 55 – 75 years of age and over (n=2103). | Unpartnered older adults are at a greater risk of problem gambling due to gambling out of loneliness. Widowed men and women had higher PG scores and were significantly more likely to gamble due to loneliness. Women who were widowed and gambled to socialise had lower PG scores. Divorced older adults, and in particular women had the highest PG scores. | *Future public health action*: Treatment and prevention of PG for older adults needs to address social isolation and loneliness, and grief and loss. | None declared. |
| Hagen, B, Nixon, G, & Solowoniuk, J. (2006). | *Aim:* To present the results of a qualitative phenomenological study regarding older adults and non-problem gambling.<br><br>*RQs:* no research question. | Individual Socio-cultural Environmental | Canada | Exploratory, phenomenological-hermeneutic study including qualitative in-depth interviews. | 60 - 68 years old, non-problem gamblers (n=12). | Older adults gambled to be included being social, for the food at the venue, for the excitement, giving to charity, inexpensive holiday and safe way to be 'bad'. Older people often went to gambling venues for other activities and gambling. Participants managed their gambling with had a range strategies. Most older people identified gambling as a risky past time. However, gambling is a social activity for this age group. | *Future research*: more research to further investigate older adults and gambling.<br><br>*Future health promotion initiatives*: To educate older adults about strategies to minimise harm from gambling. | None declared. |

| Authors | Aims and Research Questions | Determinants of gambling investigated | Country | Study Methodology | Sample | Key findings | Recommendations | Funding Sources |
|---|---|---|---|---|---|---|---|---|
| Hillbrecht, M, & Mock, S. E. (2019). | *Aim:* This study aimed to examine outcomes experienced by older adults who gamble at recreational or low to moderate risk levels to understand potential protective factors.<br><br>*RQs*: No research question. | Individual, socio-cultural | Canada | Cross sectional quantitative. | 55 years and over, (n = 3232). | This study found that the older adults who gamble at recreational levels have greater role complexity and a wider range of leisure activities and this was associated with greater social support and less stress. The results suggest that for LMR gamblers, maintaining a range of social roles and leisure activities may enhance quality of life. | *Future research:* More research about the social nature of gambling activities pursued by older adults would increase understanding of gambling as a social activity.<br>*Future public health action:* Greater attention could be given to the range of leisure activities available for older adults. | No conflicts of interest declared by authors. No funding source declared. |
| Hope, J, & Havir, L. (2002). | *Aim*: To explore and describe the meaning of casino gambling for older adults.<br><br>*RQs*: No research question. | Individual<br>Socio-cultural<br>Environmental<br>Commercial | United States | Exploratory two stage mixed methods study including a mail survey and in-depth interview. | 60 - 85+ years old, survey (n=146), interview (n=22). | Older adults attended gambling locations as they felt safe, to engage in social activities or incentives offered. Protective strategies were used to prevent overspending. Older adults did not see themselves as problem gamblers or vulnerable for gambling harm. Most were supportive of legislative changes to restrict gambling. | *Future research*: To examine lifetime patterns of gambling and compare age groups within the older adult category. | None declared. |

| Authors | Aims and Research Questions | Determinants of gambling investigated | Country | Study Methodology | Sample | Key findings | Recommendations | Funding Sources |
|---|---|---|---|---|---|---|---|---|
| Kim, W. (2020). | *Aim:* An exploration of the perceptions, beliefs, values, motivations and behaviours related to mahjong for older Chinese immigrants in New York City.<br><br>*RQs*: No research question. | Individual, Socio-cultural, Environmental | United States | Qualitative secondary data analysis. | Older Chinese immigrant, 65 years and over (n=14). | Older Chinese adults perceived mahjong as a way to stay healthy due to social connection, improving cognitive health and as a way to pass time.<br>In terms of gambling, older adults indicated that only small amounts were gambled, which justified it as a lower risk activity. Participants also stated winning wasn't the goal, but it was more enjoyable with money and with winning. However, participants also stressed they did not have routines surrounding their mahjong. | *Future research:* A comparison of Chinese immigrants living in two different environments to further understand the impact of environmental and individual factors to play mahjong.<br><br>*Future public health action:* Senior centres frequented by older Chinese immigrants my need to develop educational programs about being physically active, problem gambling and available services. | Partially funded by Fahs-Beck Fund for Research and Experimentati on. |

| Authors | Aims and Research Questions | Determinants of gambling investigated | Country | Study Methodology | Sample | Key findings | Recommendations | Funding Sources |
|---|---|---|---|---|---|---|---|---|
| Kim, W, & Kim, S. (2020). | *Aim:* The study aimed to explore the gambling behaviours and beliefs of older Korean immigrants living in New York City.<br><br>*RQs*: No research question. | Individual, socio-cultural, environmental | United States | Qualitative study. | Korean immigrant 65 years and older (n=20). | Korean immigrants shared negative perceptions about gambling retained from their culture of origin, however, they had adapted to the norms of their current environment and used gambling as a leisure or coping strategy. | *Future public health action:* There is a need for healthy, affordable and culturally appropriate interventions to assist problem gamblers. | Fahs-Beck Fund for Research and Experimentati on. |

| **Authors** | **Aims and Research Questions** | **Determinants of gambling investigated** | **Country** | **Study Methodology** | **Sample** | **Key findings** | **Recommendations** | **Funding Sources** |
|---|---|---|---|---|---|---|---|---|
| Lelonek – Kuleta, B. (2021).<br><br>Title: Gambling and retirement A qualitative study of non-problem gambling in the life course of women. | *Aim:* This study aimed to explore older women's experiences of gambling, including their gambling activity and the role gambling plays in their lives after retirement.<br><br>*RQs*: No research question. | Individual, Socio-cultural | Poland | Qualitative – in depth interviews. | Females aged 55 years and older (n=26). | Retired women with a long history of gambling increased their frequency of gambling upon retirement while those who are new to gambling gambled more intensely when they started, however, this seemed to moderate due to financial constraints. The increase in free time seems to impact increases in gambling frequency. | *Future public health action:* Older women need to be supported in retirement to access recreational activities that are beneficial. Older women need to be educated about gambling products. Gambling policy needs to promote controlled gambling. | None declared. |

| Authors | Aims and Research Questions | Determinants of gambling investigated | Country | Study Methodology | Sample | Key findings | Recommendations | Funding Sources |
|---|---|---|---|---|---|---|---|---|
| Lelonek – Kuleta, B. (2022).<br><br>Title: Gambling motivation model for older women addicted and not addicted to gambling – a qualitative study. | *Aim: The* study aimed to understand the motivations of professional older women addicted and not addicted to gambling.<br><br>*RQs*: No research question. | Individual, Socio-cultural | Poland | Qualitative study. | 55 years and over women (n=34). | Five categories of motivation were identified: sensations, money, activity, socialisation and escape. Women who were not addicted to gambling were motivated due to sensations, money and activity and socialisation. Women who were addicted to gambling were mostly motivated by sensations, escape and socialisation. | *Future public health action:* Building social networks for older adults. Ensure older adults are aware of the risks and odds of winning when gambling, and where to seek help. | Ministry of Health. |

| **Authors** | **Aims and Research Questions** | **Determinants of gambling investigated** | **Country** | **Study Methodology** | **Sample** | **Key findings** | **Recommendations** | **Funding Sources** |
|---|---|---|---|---|---|---|---|---|
| Lelonek – Kuleta, B. (2022). | *Aim:* Learn about the motivation to gamble for retired Polish male seniors.<br>*RQs:* What are the gambling motives among Polish retired men? What categories of gambling motives are most popular among retired men? Does the motivation of retired men to gamble vary with the severity of problem gambling? | Individual<br>Socio-cultural | Poland | Qualitative. | Retired men who gamble aged 55 – 83 (n=44). | Motivational factors common to male senior gamblers were self-gratification such as gambling for entertainment or seeking positive sensations and that the need for these positive experiences is related to life satisfaction and wellbeing. For non-problem gamblers, social motivations drew them to gamble while for problem gamblers, their motivations were mostly for financial reasons or to cope with difficult situations. | *Future research:* Quantitative research on Polish seniors to understand psychological mechanisms underlying development of gambling problems. | Ministry of Health in Poland, Gambling Problem Solving Fund awarded to The John Paul II Catholic University of Lublin. |

| Authors | Aims and Research Questions | Determinants of gambling investigated | Country | Study Methodology | Sample | Key findings | Recommendations | Funding Sources |
|---|---|---|---|---|---|---|---|---|
| Loroz, P.S. (2004). | *RQs:* Why is gambling so popular among senior consumers? What psychological benefits does the gambling experience provide to members of this group? | Individual Socio-cultural Environmental | United States | Qualitative phenomenological investigation. | 55 – 82 years of age (n=27), gamblers who participated in games of chance at a casino. | Three main themes were identified in this study relating to older adults experiences of gambling. Older adults gambled for control: by making choices, changing their luck, acknowledging loss probabilities, with spending limits and with their reactions to their experiences. Gambling provided a lift for older adults: physically, emotionally and for sensory experiences. Additionally gambling provided older adults an escape: from regular routines, to regain social contact, with money as entertainment and fantasising about winning. The authors identified fun, fantasy and feelings as drawcard for older adults to begin gambling with these themes of control, lift and escape being intermediary psychological responses that may impact on older adults self concept. | *Future research:* Future research could test the proposed model related to self-concept dynamics, and explore the self-concept dynamic with other experiential consumption activities with a range of consumer groups. | None declared. |

| Authors | Aims and Research Questions | Determinants of gambling investigated | Country | Study Methodology | Sample | Key findings | Recommendations | Funding Sources |
|---|---|---|---|---|---|---|---|---|
| Luo, H. (2021). | *Aim:* The study aimed to understand older Chinese gamblers through a life course perspective.<br><br>*RQs*: No research question. | Individual, socio-cultural, environmental | Canada | Qualitative, in depth interviews. | Chinese-Canadians 60 years or older (n=15). | Gambling was common in participants childhood, and participants gambled to socialise, due to loneliness or to escape negative life circumstances or past trauma. Older adults reported casinos as convenient, safe places with free food and drinks. | *Future public health action:* Policymakers and practitioners may need to focus on facilitation of socialisation for older adults. Consider the quality of social interactions older adults receive from gambling and whether there are more appropriate ways to support older adults to socialise. | Manitoba Gambling Research Program Grant. |

| Authors | Aims and Research Questions | Determinants of gambling investigated | Country | Study Methodology | Sample | Key findings | Recommendations | Funding Sources |
| --- | --- | --- | --- | --- | --- | --- | --- | --- |
| McCarthy, S, Pitt, H, Bellringer, M.E. & Thomas, S.L. (2022). | *Aim:* This study explored how individual and socio-cultural factors lead to EGM gambling in older women, whether gambling environments impact older women's gambling and if older women describe any commercial determinants regarding their gambling. | Individual, Socio-cultural, Environmental, Commercial | Australia | Qualitative constructivist grounded theory approach. | 55 years and over, women (n=20). | Older women used EGM Gambling to escape stress or negative life situations, in particular key events. Older women used EGM venues to fill void of feeling lonely, however with increased gambling led to further isolation. EGM venues considered safe and inclusive for older women, and factors about venues made women stay for longer (ie; free food and drinks). | *Future research:* Incorporate key commercial and political determinants of older women's' gambling.<br><br>*Future public health action:* Interventions need to be aimed at providing alternative venues to meet older women's' needs, including withdrawing commercial factors that make gambling venues appealing options. | Funding for study not specifically listed, funding for each researcher is listed and there is a range. |
| Martin, F, Lichtenberg, P.A. & Templin, T.N. (2011). | *Aim*: To describe the demographic characteristics, frequency of gambling, attitudes towards and motivations for gambling at casino.<br><br>*RQs:* No research question. | Individual Socio-cultural Environmental | United States | Secondary data analysis of two quantitative surveys. | 60 years and older, (n=247). | Participants generally held positive views of casino gambling. Older adults went to casinos for entertainment, to win, to escape feelings of grief, boredom and loneliness. | *Future research:* examination of health issues associated with gambling. | None declared. |

| Authors | Aims and Research Questions | Determinants of gambling investigated | Country | Study Methodology | Sample | Key findings | Recommendations | Funding Sources |
|---|---|---|---|---|---|---|---|---|
| McNeilly, D. P, & Burke, W. J. (2000). | *Aim*: To investigate the prevalence of gambling behaviour, depression, life satisfaction and motivations for gambling.<br><br>*RQs*: No research questions. | Individual Socio-cultural | United States | Cross sectional self-report survey. | 67 to 78 years old, (n=315). | Those sampled at gambling venues were more likely to have disordered gambling, more frequent spending on a number of forms of gambling, increased spending on gambling, smoke, drive a car, eat fewer than two meals per day and do occasional volunteer work. For all older adults, motivations for gambling included relaxation, boredom, passing time and getting away for the day.<br>Both groups (gambling patrons vs community group) had various levels of 'problem' and 'probable pathological gambling'.<br>Gambling patron group more likely to report they had gambled more than they meant to and that gambling was harmless fun.<br>Most often play slot machines at casinos. | *Future research*: To conduct a representative study of gambling and older adults. | None declared. |

| Authors | Aims and Research Questions | Determinants of gambling investigated | Country | Study Methodology | Sample | Key findings | Recommendations | Funding Sources |
|---|---|---|---|---|---|---|---|---|
| Ng, V.C.K. (2011). | *Aim*: To provide some understanding of older adults gambling in Singapore.<br><br>*RQs:*<br>Five research questions explored gambling participation and preferred method of gambling, perceptions of gambling, planned behaviour regarding casinos and help seeking knowledge. | Individual<br>Socio-cultural<br>Environmental | Singapore | Exploratory quantitative study. | 60 - 91 years old, (n=74). | On average, older adults gambled twice a week, spending $12. Gamblers had significantly more 'free time' than non-gamblers. Lottery was the most popular type of gambling. Those with lower incomes spent greater proportions of their incomes on gambling. While there were positive perceptions of gambling, casinos were seen negatively.<br>Most participants (97%) did not know how to get help for gambling issues. | *Future public health action:* Social workers and other groups need to collaborate to ensure they reach isolated older adults.<br>*Future health promotion:* Public education campaigns are needed to ensure they reach older adults. | The Ang Mo Kio Family Service Centres (AMKFSC). |

| **Authors** | **Aims and Research Questions** | **Determinants of gambling investigated** | **Country** | **Study Methodology** | **Sample** | **Key findings** | **Recommendations** | **Funding Sources** |
|---|---|---|---|---|---|---|---|---|
| O'Brien Cousins, S, & Witcher, C. (2004). | *Aim*: No aim.<br><br>*RQs:* What is the essential nature and meaning of being a bingo player? What do older women 'get' out of playing bingo? How do they get involved in the game? How do bingo stigmas and stereotypes affect them? What contribution does bingo offer to aging well and quality of life, if any? In what ways may bingo alter health risks of older women? | Individual<br>Socio-cultural | Canada | Inductive qualitative approach using personal experience, interview, observation, field notes and social interactions. | 66 - 87-year-old female participants (non-problem bingo players) (n=8) interviews and (n=9) in focus group. | Motivations for playing bingo included feeling good around other people, winning money, helping the community, that Bingo was easy to participate in, and to have fun.<br>Perceptions around winning and losing varied - some enjoy the game as a game, others need to budget to play. There was a perception that it was increasingly more difficult to win money at Bingo.<br>Some participants felt like the stigma of gambling/bingo impacted on them. | *Future research*: Further research could be conducted on male bingo players, and why some women don't want men playing Bingo. | Alberta Gaming Research Institute. |

| **Authors** | **Aims and Research Questions** | **Determinants of gambling investigated** | **Country** | **Study Methodology** | **Sample** | **Key findings** | **Recommendations** | **Funding Sources** |
|---|---|---|---|---|---|---|---|---|
| O'Brien Cousins, S, & Witcher, C. S. G. (2007). | *Aim:* To explain the social context and lifestyle of senior bingo players.<br>*RQs*: "(1) Who plays bingo in late life (age 65+); how do bingo players differ in social and health characteristics from non-players?<br>(2) What are the important lifestyle patterns of seniors who play bingo? (3) How accurate are the gambling stigmas and social stereotypes for the game of bingo?" | Individual<br>Socio-cultural<br>Commercial | Canada | Two stage cross sectional quantitative study. | 65 years and older, stage 1 (n=400) stage 2 (n= 44) bingo players. | Participants considered gambling an affordable way to have time out. Females, more elderly, renting, receiving income supplementation, more reported health problems, being sedentary and widowed more likely to spend more money on bingo. Bingo players more likely to spend money on other gambling activities. | *Future public health action:* Public health messaging around recreational activities needs to be reviewed as currently it is not having an impact. | None declared. |

| Authors | Aims and Research Questions | Determinants of gambling investigated | Country | Study Methodology | Sample | Key findings | Recommendations | Funding Sources |
|---|---|---|---|---|---|---|---|---|
| Ohtsuka, K and Chan, C.C. (2014). | *Aim:* To understand the place of gambling for older adults in HK.<br><br>*RQs*: no research question. | Individual Socio-cultural | Hong Kong | Mixed methods study with an ethnographic approach. Involved a survey and semi-structured interviews. | 55-85 years old, (n=18). | Participants were introduced to gambling by family members and began gambling at a young age. Most gamblers gambled for social reasons. Female gamblers tended to gamble for social reasons and fun and males more often to win. Gender differences were observed in gambling activities with males preferring card games, horse and sport betting while women mostly played Mahjong. 3 participants were classified as PG. Participants indicated they were 'playing' when gambling and that gambling was normalised for them. | No recommendations. | None declared. |
| Parekh, R ,& Morano, C. (2009). | *Aim:* To understand reception of a gambling education program and to understand participants behaviour and attitudes towards gambling.<br><br>*RQs*: no research questions. | Individual Sociocultural | United States | Sequential exploratory mixed methods study with qualitative preceding quantitative. | 60 to 80-year olds, (n=137). | Older adults are gambling because they have no other forms of recreation, for excitement, fun and to try to make money back.<br>While gambling itself appeared to hold less stigma, having a gambling problem was highly stigmatised. | *Future research*: Conduct research to understand how gambling is impacting minority groups of older adults.<br>*Future public health action*: Education of older adults, social service and health care professionals needs to occur regarding potential issues caused by gambling. | None declared. |

| Authors | Aims and Research Questions | Determinants of gambling investigated | Country | Study Methodology | Sample | Key findings | Recommendations | Funding Sources |
|---|---|---|---|---|---|---|---|---|
| Parke, A, Griffiths, M, Pattinson, J, & Keatley, D. (2018). | *Aim: To* "assess the association between age-related physical health, social networks, and problem gambling in adults aged over 65 years and to assess the mediating role of affective disorders in this association." *RQs:* No research question. | Individual Socio-cultural Environmental | United Kingdom | Cross sectional quantitative study that included a survey that assessed physical frailty, geriatric pain, loneliness, geriatric depression, geriatric anxiety, and problem gambling. | 65 - 94 years old, (n=595). | Late-life problem gambling may develop as gambling is used as an 'escape' from anxiety and depression and these states are often due to deteriorating physical wellbeing and social support. Gambling venues are a safe, accessible location for older adults. | *Future research:* Investigate if older adults with physical frailty use gambling to modulate mood. | Responsible gambling trust. |

| Authors | Aims and Research Questions | Determinants of gambling investigated | Country | Study Methodology | Sample | Key findings | Recommendations | Funding Sources |
|---|---|---|---|---|---|---|---|---|
| Pattinson, J, & Parke, A (2018). | *Aim:* To identify and explore motivations for gambling in high frequency older adult female gamblers in the United Kingdom.<br><br>*RQs:* no research question. | Individual Socio-cultural Environmental | United Kingdom | Interpretive phenomenological analysis using semi structured interviews. | Females 60 - 80 years of age, (n=10). | Motivations for gambling included filling voids and emotional escape. Many participants overspent their budgeted amount while gambling and felt the environment led to this. There was a perception that problem gambling is not an issue later in life. | *Future research:* Investigate potential risk factors unique to this population. | PhD bursary, awarded to JP from the Responsible Gambling Trust.<br><br>Declaration of interest. AP occasionally acts as a paid responsible gambling consultant for both offline and online gambling operators, including state-run gambling industries. |

| **Authors** | **Aims and Research Questions** | **Determinants of gambling investigated** | **Country** | **Study Methodology** | **Sample** | **Key findings** | **Recommendations** | **Funding Sources** |
|---|---|---|---|---|---|---|---|---|
| Pattinson, J, & Parke, A (2016). | *Aim:* To provide a framework of older adults cognitive and behavioural patterns.<br>*RQs:* no research question. | Individual<br>Socio-cultural<br>Environmental | United Kingdom | Qualitative. | 69 – 92 year olds, mean age of 76.82 (n=17). | Four theoretical propositions from the research are: gambling has limited deterrents to participation for older adults, including it being an available and accessible pastime when compared with other recreational activities; gambling provides a temporary escape from psychological distress associated with aging including loneliness, bereavement and retirement; gambling provides a distraction from physical pain and negative affect of related physical deterioration; gambling meets psychological needs for pleasure and mental stimulation. | *Future research:* Investigate to determine if findings from this study are applicable across other groups of older adults to begin to understand the specific vulnerabilities of older adults. This information can then inform prevention, screening and intervention for older adults. | Funded by a research scholarship from the Responsible Gambling Trust (RGT). |
| Penalba, E.H. (2020). | *Aim:* Explore cockfighting as a form of gambling.<br>*RQs:* no research question. | Individual<br>Socio-cultural<br>Environmental | Philippines | Qualitative | Males 80 year olds and over (n=2). | Themes reported from this study included: cockfighting as an all-consuming pastime, as a thrill seeking activity, family influence on participation in cockfighting, fighting venues as a site for socialisation, betting to earn money, and gambling as a family issue. | No recommendations. | None declared. |

| Authors | Aims and Research Questions | Determinants of gambling investigated | Country | Study Methodology | Sample | Key findings | Recommendations | Funding Sources |
|---|---|---|---|---|---|---|---|---|
| Phillips, W. J, & Jang, S. (2012). | *Aim:* To investigate whether attitude, subjective norms, perceived behavioural controls along with motivation influence seniors casino patronage intentions differently based on if they perceive themselves as regular or irregular casino patrons".<br><br>*RQs:* No research questions | Individual | United States | Quantitative online survey-based study. | 65 years old or older, (n=681). | Seniors winning and enjoyment significantly influenced casino gaming intentions, past casino experience did not moderate effects of the predictors (attitude, subjective norm, perceived behavioural control and motivation) on future intention.<br>All predictable variables of the theory of planned behaviour positively affected casino intentions. An outcome of the study was an extended version of the theory of planned behaviour to help predict seniors gambling behaviour. | *Gambling industry recommendations:* Made recommendations to the gambling industry of how to bring in more patrons. Eg; more promotions and more frequent, smaller wins. | None declared. |

| Authors | Aims and Research Questions | Determinants of gambling investigated | Country | Study Methodology | Sample | Key findings | Recommendations | Funding Sources |
|---|---|---|---|---|---|---|---|---|
| Pitt, H, Thomas, S. L, Cowlishaw, S, Randle, M, & Balandin, S. (2022). | *Aim:*to understand older adults motivations, knowledge and perception of risk relating to EGM use. | Individual<br>Environmental | Australia | Qualitative study with data collected through focus groups. | 55 and over, (n= 126). | Participants' attended community gambling venues for non gambling reasons, however, often engaged with EGMs due to their availability. Some older adults gambled to zone out or escape stress.<br>Some participants identified factors that venues used to encourage OA to use EGMs, they felt they were not at risk due to their RG strategies (eg: setting time and money limits).<br>Older adults demonstrated misconceptions about how EGM worked (including how often they will pay out). Findings suggest that OA may be less receptive to RG messages. | *Future research:* Research that explores older adults receptivity to information about gambling products, including EGMs and their design and the associated harms.<br><br>*Future public health action:* Reduce availability and accessibility of EGMs in Australian communities. Develop public education messaging about gambling products that are appropriate for older adults. | Funding for study not specifically listed, funding for each researcher is listed and there is a range. |

| Authors | Aims and Research Questions | Determinants of gambling investigated | Country | Study Methodology | Sample | Key findings | Recommendations | Funding Sources |
|---|---|---|---|---|---|---|---|---|
| Southwell, J, Boreham, P, Laffan, W. (2008). | *Aim:* investigate personal characteristics that influence older adults EGM behaviour and motivation, and if venue promotions impact on their behaviour and motivation.<br><br>*RQs:* No research questions. | Individual<br>Socio-cultural<br>Environmental | Australia | Mixed methods study survey of EGM users. | 60 years and older EGM users, (n=414). | Older adults were gambling to support club (46%), win (45%), be social (41%), decrease boredom (39%), decrease isolation (34%) and decrease depression/stress (33%). Higher levels of motivation for gambling are associated with those who have no partner, disability, low income, no longer in workforce. EGMs used to meet social, health and recreational needs. Younger (60-69 y.o.), male, single participants more likely to be motivated by excitement and winning. Older adults experienced betting outside self determined limits, reduced control over session length, using savings to finance gambling and holding erroneous beliefs about the outcome of the machines. | *Future research:* Investigate the impact of venue promotions on older adults gambling and potential policy action. | Research grant from the Queensland Office of Gaming Regulation an agency of the Queensland State Government. |

| Authors | Aims and Research Questions | Determinants of gambling investigated | Country | Study Methodology | Sample | Key findings | Recommendations | Funding Sources |
|---|---|---|---|---|---|---|---|---|
| Subramaniam, M, Chong, S. A, Satghare, P., Browning, C. J, & Thomas, S (2017). | *Aim:* To explore both the role families play in gambling initiation, maintenance, and help-seeking, and the harm caused to families by the gambling behaviour using a qualitative approach.<br><br>*RQs*: No research question. | Individual<br>Socio-cultural | Singapore | Qualitative | 60 years or older, mean age of 66.2 years, (n=25). | The majority of participants were exposed to gambling at a young age and considered it a normal, social activity. Some used gambling as an escape (mostly for women). Gambling related harm experienced by participants included financial problems, relationship problems and family breakdown. Participants hid problem behaviour, and families threatened older adults if they didn't get help. The stigma of problem gambling was a barrier to help seeking. | *Future research:* Interventional studies for reducing stress and improving coping among family members.<br><br>*Future public health action:* Education of family members to encourage help-seeking to ensure early treatment and recovery. | Ministry of Health, National Medical Research Council (NMRC). |
| Subramaniam, M, Satghare, P., Vaingankar, J. A., Picco, L, Browning, C. J, Chong, S. A, & Thomas, S (2017). | *Aim:* To describe the concept of RG among older adults, and the cognitive and behavioural strategies employed by older adults. | Individual<br>Socio-cultural | Singapore | Qualitative | 60 years or older, mean age of 66.2 years (SD=6.5), (n=25). | The key themes identified from this study were 1) self-developed strategies to limit gambling related harm, and 2) family interventions to decrease gambling harm. | *Future research:* Longitudinal study to determine the effectiveness of RG strategies implemented by older adults. Other research examining effectiveness of family invoked exclusion, and gambling operator initiatives to encourage RG.<br>*Future public health action:* Education of both family members and older adults in RG behaviours. | Ministry of Health, National Medical Research Council (NMRC). |

| Authors | Aims and Research Questions | Determinants of gambling investigated | Country | Study Methodology | Sample | Key findings | Recommendations | Funding Sources |
|---|---|---|---|---|---|---|---|---|
| Thériault, É. R, Norris, J. E, & Tindale, J. A. (2018). | *Aim:* To determine the rates of problem gambling within the study and to examine responsible gambling strategies being used and their effectiveness.<br><br>RQs: No research questions. | Individual | Canada | Secondary data analysis of quantitative data. | 55 years and older, (n=673). | Limiting money spent was the most frequent Responsible Gambling strategy used. There was no evidence that responsible gambling strategies were related to risk of problem gambling in older adults. Those who used responsible gambling strategies did not have a lower problem gambling risk than those who did not use strategies. | *Future research:* To understand which Responsible Gambling strategies are appropriate and effective for older adults. | Gambling Research Exchange Ontario. |
| Tira, C, & Jackson, A. C. (2015) | *Aim:* To explore the perceptions of gambling in senior gamblers aged 55 years and older who reside in Victoria, Australia.<br><br>*RQs:* No research questions. | Individual | Australia | Secondary analysis of qualitative data. | 56 to 85-year olds, (n=31). | Definitions of gambling varied and depended on the individual. Gambling activities tended to be entertaining and involved luck. Activities were not considered gambling when the activity is part of their identity, the cost is comparable to other leisure activities, when it occurs alongside other activities or events, if it is not your money, when the experience doesn't 'feel real' and when the venue feels like home. | *Future research:* To ask about particular gambling activities rather than use gambling as a term to determine behaviour. Future research should not assume a common understanding of definition of gambling when conducting public health campaigns or screening for gambling problems. | None declared. |

| **Authors** | **Aims and Research Questions** | **Determinants of gambling investigated** | **Country** | **Study Methodology** | **Sample** | **Key findings** | **Recommendations** | **Funding Sources** |
|---|---|---|---|---|---|---|---|---|
| Turner, N.E, van der Maas, M, McCready, J, Hamilton, H.A, Schrans, T, Ialomiteanu, A, Ferentzy, P, Elton-Marshall, T, Zaheer, S, & Mann, RE, (2018). | *Aim:* To understand the gaming and gambling habits of older adults at gambling venues.<br><br>*RQs*: Six research questions explored patterns of gambling behaviour, gambling expenditure, rates of problem gambling, problem gambling in relation to proximity to casino, gambling problems experienced in relation to bus tours and the impact of a fixed income on gambling problems. | Individual<br>Environmental<br>Commercial | Canada | Cross sectional self-report survey and quantitative analysis. | 55 to 75 and older gamblers, (n=2103). | Participants gambled on 3.6 different activities in past 12 months, 78.6% used EGMs. 6.9% indicated severe problem gambling on PGSI with another 20.3% having moderate gambling problems. Gambling activities included- EGMs, instant win/lotteries, sport lotteries, horse racing, bingo, other casino games, card, board games and low rates of online activities. Those who went on bus tours more likely to have higher scores on PGSI. | *Future research:* Further research is required on the needs of the age group, and methods to reduce gambling harm for this age group.<br><br>*Future public health action:* Findings demonstrate a need for change to gambling policy. Recommendation that information on gambling harm is provided on casino courtesy buses. | Ontario Problem Gambling Research Centre and Ministry of Health and Long-Term Care. |

| Authors | Aims and Research Questions | Determinants of gambling investigated | Country | Study Methodology | Sample | Key findings | Recommendations | Funding Sources |
|---|---|---|---|---|---|---|---|---|
| Van der Maas, M, Hamilton, H.A, Matheson, F.I, Mann, R.E, Turner, N. E, and McCready, J. (2019). | This study investigated the associations between motivations to gamble at a casino and problem gambling. | Individual | Canada | Cross sectional survey. | 55 years and older (n= 2103). | Seeking excitement, socialisation and avoiding boredom were the most frequently endorsed motivations by men and women. Motivations related to money and emotion were associated with higher odds of PG. Motivations related to entertainment were associated with lower odds of PG. The association between emotional motivations and PG in men was significantly stronger than that with females. | *Future research:* More research is required to compare these gambling behaviours of older adults to younger cohorts and whether there are reduced gender differences in gambling as a whole.<br><br>*Future public health action:* Responsible gambling provisions should discourage those who are vulnerable to gambling. | Supported by Gambling Research Exchange Ontario and the Centre for Addiction and Mental Health. |
| van der Maas, M, Mann, R. E, Matheson, F. I, Turner, N. E, Hamilton, H. A, & McCready, J. (2017). | *Aim:* To determine if casino bus tour attendees have high odds of being problem gamblers.<br><br>RQs: No research questions. | Individual Environmental | Canada | Cross sectional quantitative study. | 55 to 75 and older gamblers, (n=2103). | Bus tour patronage associated with higher odds of problem gambling, type of gambling and gambling expenditure. Additionally, being separated or divorced led to a higher rate of problem gambling. Bi variate analysis demonstrated that past year bus tour patronage associated with more frequent slot machine play, more past year gambling venue visits and lower spending on gambling per casino visit.<br>Bus tour patrons more likely to be Female, born outside of Canada, 75 years and retired. | *Future public health action:* Regulations need to be considered regarding vulnerable populations accessing casinos | Gambling Research Exchange Ontario. |

| **Authors** | **Aims and Research Questions** | **Determinants of gambling investigated** | **Country** | **Study Methodology** | **Sample** | **Key findings** | **Recommendations** | **Funding Sources** |
|---|---|---|---|---|---|---|---|---|
| van der Maas, M, Mann, R. E, Turner, N. E, Matheson, F. I, Hamilton, H. A, & McCready, J. (2018). | *Aims:* To examine prevalence of gambling and gambling problems, gender differences around gambling behaviour and specific types of behaviour related to gambling.<br><br>*RQs:* No research questions. | Individual<br>Socio-cultural<br>Commercial | Canada | Secondary data analysis of quantitative survey. | 55 and older, (n=2187). | Participants were more likely to gamble if they were divorced, single, and separated. EGMs, lottery and scratch tickets were the most popular gambling activities. Problem gamblers made up 7.6% of the sample and were more likely to participate at least monthly in a range of gambling activities. | *Future research:* Qualitative research that provides older adults the opportunity to talk about motivations and experiences and how best to reach them. | Ontario Ministry of Health and Long Term Care, Health System Research Fund (Grant No. 06701). The project was sponsored and administered by the Centre for Addiction and Mental Health (CAMH). |

| **Authors** | **Aims and Research Questions** | **Determinants of gambling investigated** | **Country** | **Study Methodology** | **Sample** | **Key findings** | **Recommendations** | **Funding Sources** |
|---|---|---|---|---|---|---|---|---|
| Venuleo, C, Marinaci, T, & Mossi, P. (2021). | The study aimed to examine Italian older adults gambling, and the role of loneliness, social support and well being in describing their problem with gambling. | Individual, socio-cultural | Italy | Cross sectional study. | 60 years and over (n=165). | Older adults gambled to win money and forget their problems and generally older adults gambled the most on the lottery or scratch tickets.<br>Nearly 1/3 of participants indicated they would not seek help for the gambling.<br>Moderate risk/problem gamblers were more likely to have increased perceived loneliness scores and lower perceived social support and well-being. | *Future research:* Address the psychosocial determinants of gambling which are influenced by cultural and structural drivers.<br><br>*Future public health action:* Prevention of gambling harm should include addressing the interpersonal and social level. Community organisations not related to gambling need to address the social needs of older adults. | Not listed. |
