# Supplementary material for: A scoping review of the individual, socio-cultural, environmental and commercial determinants of gambling for older adults: implications for public health research and harm prevention
Source: BMC Public Health. 2023 Feb 20;23:362. doi: 10.1186/s12889-022-14930-y (PMC9940406; doi:10.1186/s12889-022-14930-y)
Supplement: Supplementary file 3 — Additional file 3: Supplementary Table S3. Table of papers excluded at full text, with reasons. [file 12889_2022_14930_MOESM3_ESM.pdf]

Supplementary Table S3: Table of papers excluded at full text, with reasons.

Authors and Journal	Exclusion Reason
Baker, J. C. (2010). Behavioural Gerontology and Gambling: The Jackalope of Behaviour Analysis. Analysis of Gambling Behavior, 4, 1, 5 – 15.	Literature review study.
Bjelde, K., et al. (2008). "Casino gambling among older adults in North Dakota: A policy analysis." Journal of Gambling Studies **24**(4): 423-440.	General population sample.
Blackman, A., Browne, M., Rockloff, M., Hing, N and Russell, A.M.T. (2019). Contrasting Effects of Gambling Consumption and Gambling Problems on Subjective Wellbeing. Journal of Gambling Studies, 5, 3, 773. DOI: 10.1007/s10899-019-09862-z	General population sample.
Chen, R. J. and Dong, X. Q. (2015). The Prevalence and Correlates of Gambling Participation among Community-Dwelling Chinese Older Adults in the U.S. Aims Medical Science, 2, 2, 90-103.	Demographic characteristics.
Ciofi, J. (2020). The Ambivalent Subject: Reconciling Contradictory Subjective Experiences of Surveillance. Surveillance and Society, 18, 1, 61 – 73.	General population sample.
Clarke, D. (2008). "Older adults' gambling motivation and problem gambling: A comparative study." Journal of Gambling Studies **24**(2): 175-192.	General population sample.
Clarke, D. and Clarkson, J. (2009). "A preliminary investigation into motivational factors associated with older adults' problem gambling." International Journal of Mental Health and Addiction **7**(1): 12-28.	Problem gambling study.
del Pino-Gutierrez, A.<br>Granero, R.<br>Fernandez-Aranda, F. et al (2021). Gambling activity in the old age general population. *Ageing and Society*. https://doi.org/10.1017/S0144686X21000258	Demographic characteristics.
Depping, M.K., Best, R. and Freund, A.M. (2021). From gains to losses: Age – related differences in decision under risk in non-monetary gambling task. British Journal of Developmental Psychology, 39, 2, 312-329.	General population sample.
Erickson, L., et al. (2005). "Problem and pathological gambling are associated with poorer mental and physical health in older adults." International Journal of Geriatric Psychiatry **20**(8): 754-759.	Problem gambling study.

Ferland, F., et al. (2006). "Characteristics of gamblers taking chartered day trips to casinos." Canadian Journal of Community Mental Health **25**(1): 67-73.	Demographic characteristics.
Fiedor, D., Krai, O., Frajer, J., Sery, M and Szczyrba, Z. (2019). What do Residents Consider to be Gambling and What are Their Attitudes Towards it? Evidence from the Czech Republic. Journal of Gambling Studies, 35, 1347 – 1360. Doi: 10.1007/s10899-018-9804-9	General population sample.
Gainsbury, S. M., et al. (2018). "Strategies to customize responsible gambling messages: a review and focus group study." BMC Public Health **18**(1): 1381-1381.	General population sample.
Granero, R., Jiminez-Murcia, S., Pino-Gutierrez, A., Mena-Moreno, T., et al. (2020). Gambling Phenotypes in Older Adults. Journal of Gambling Studies, 36, 809 – 828. Doi: 10.1007/s10899-019-09922-4	Demographic characteristics.
Granero, R., Jiminez-Murcia, S., Fernandez-Aranda, F., Pino-Gutierrez, A., Mena-Moreno, T., et al. (2020). Presence of problematic and disordered gambling in older age and validation of the South Oaks Gambling Scale. PloS one, 15, 5, e0233222. Doi: 10.1371/journal.pone.0233222	Screening tool.
Granero, Roser<br>Jiménez-Murcia, Susana<br>Fernández-Aranda, Fernando et al (2022). Contribution of stressful life events to gambling activity in older age. Ageing and Society, 42, 1513 – 1537.	Problem gambling study.
Heiskanen, M.K. and Matilainen, R. (2020). Baby boomers as gamblers: recognising and preventing gambling harm with intersectional approach. Public Health, 184, 5 – 10. Doi:10.1016/j.puhe.2020.04.020	Demographic characteristics.
Hirshorn, B. A., et al. (2007). "Factors associated with recreational gambling frequency among older adults." International Gambling Studies **7**(3): 345-360.	Demographic characteristics.
Kardos, Z., Toth, C., Boha, R., File, B and Molnar, M. (2017). Age-dependent characteristics of feedback evaluation related to monetary gains and losses. International Journal of Psychophysiology, 122, 42 – 49.	Clinical examinations.

Kausch, O. (2004). "Pathological gambling among elderly veterans." <u>Journal of Geriatric Psychiatry and Neurology</u> **17**(1): 13-19.	General population sample
Kerber, C. S., et al. (2008). "Comorbid psychiatric disorders among older adult recovering pathological gamblers." <u>Issues in Mental Health Nursing</u> **29**(9): 1018-1028.	Demographic characteristics.
Ladd, G. T., et al. (2003). "Gambling Participation and Problems Among Older Adults." <u>Journal of Geriatric Psychiatry and Neurology</u> **16**(3): 172-177.	Problem gambling study.
McCready, J., et al. (2008). "Correlates of gambling-related problems among older adults in Ontario." <u>Journal of Gambling Issues</u> **22**: 174-194.	Demographic characteristics.
McNeilly, D. P. and W. J. Burke (2002). "Disposable time and disposable income: Problem casino gambling behavior in older adults." <u>Journal of Clinical Geropsychology</u> **8**(2): 75-85.	Problem gambling study.
Medeiros, G.C., Leppink, E, Yaemi, A, Mariani, M, Tavares, H. and Grant, J. (2015). Gambling disorder in older adults: A cross-cultural perspective. Elsevier, 58, 116-121.	Problem gambling tudy.
Potenza, M. N., et al. (2006). "Characteristics of Older Adult Problem Gamblers Calling a Gambling Helpline." <u>Journal of Gambling Studies</u> **22**(2): 241-254.	Demographic characteristics.
Preston, F. W., et al. (2007). "Successful aging and gambling: Predictors of gambling risk among older adults in Las Vegas." <u>American Behavioral Scientist</u> **51**(1): 102-121.	Demographic characteristics.
Theraiult, E.R., Norris, J.E. and Tindale, J.A. (2020) Journal of Gambling Studies (2020) 36:119–139 https://doi.org/10.1007/s10899-019-09886-5	Demographic characteristics.
van der Maas, M., Matheson, F. I., Turner, N.E., Hamilton, H.A., Mann, R.E. & McCready, J. (2019). A generational comparison of problem gambling and gambling attitudes among older adult gambling venue patrons, International Gambling Studies, 19:1, 22-35, DOI: 10.1080/14459795.2018.1497071	This paper compares two generational cohorts of older adults, rather than examining determinants of gambling of older adults.
von Hippel, W., et al. (2009). "Executive functioning and gambling: Performance on the Trail Making Test is associated with gambling problems in older adult gamblers." <u>Aging, Neuropsychology, and Cognition</u>	Clinical examinations.

**16**(6): 654-670.	
Welte, J., et al. (2001). "Alcohol and gambling pathology among U S adults: Prevalence, demographic patterns and comorbidity." Journal of Studies on Alcohol **62**(5): 706-712.	General population sample
Welte, J. W., et al. (2004). "Gambling participation and pathology in the United States--A sociodemographic analysis using classification trees." Addictive Behaviors **29**(5): 983-989.	General population sample
Wiebe, J. M. D. and B. J. Cox (2005). "Problem and Probable Pathological Gambling Among Older Adults Assessed by the SOGS-R." Journal of Gambling Studies **21**(2): 205-221.	Validation of gambling tool.
